# A Comprehensive Review of Metal–Organic Framework: Synthesis, Characterization, and Investigation of Their Application in Electrochemical Biosensors for Biomedical Analysis

**DOI:** 10.3390/s22062238

**Published:** 2022-03-14

**Authors:** Zahra Dourandish, Somayeh Tajik, Hadi Beitollahi, Peyman Mohammadzadeh Jahani, Fariba Garkani Nejad, Iran Sheikhshoaie, Antonio Di Bartolomeo

**Affiliations:** 1 Department of Chemistry, Faculty of Science, Shahid Bahonar University of Kerman, Kerman 7616913439, Iran; z.dourandish2017@gmail.com (Z.D.); f.garkani95@gmail.com (F.G.N.); i_shoaie@yahoo.com (I.S.); 2 Research Center of Tropical and Infectious Diseases, Kerman University of Medical Sciences, Kerman 7616913555, Iran; s.tajik@kmu.ac.ir; 3 Environment Department, Institute of Science and High Technology and Environmental Sciences, Graduate University of Advanced Technology, Kerman 7631885356, Iran; 4 School of Medicine, Bam University of Medical Sciences, Bam 7661771967, Iran; peymanjahani1234@gmail.com; 5 Dipartimento di Fisica “E.R. Caianiello”, Università di Salerno, 84084 Fisciano, SA, Italy

**Keywords:** biomarkers, biomedical, electrochemical biosensor, metal–organic framework, pharmaceutical drugs, small biomolecules

## Abstract

Many studies have addressed electrochemical biosensors because of their simple synthesis process, adjustability, simplification, manipulation of materials’ compositions and features, and wide ranges of detection of different kinds of biomedical analytes. Performant electrochemical biosensors can be achieved by selecting materials that enable faster electron transfer, larger surface areas, very good electrocatalytic activities, and numerous sites for bioconjugation. Several studies have been conducted on the metal–organic frameworks (MOFs) as electrode modifiers for electrochemical biosensing applications because of their respective acceptable properties and effectiveness. Nonetheless, researchers face challenges in designing and preparing MOFs that exhibit higher stability, sensitivity, and selectivity to detect biomedical analytes. The present review explains the synthesis and description of MOFs, and their relative uses as biosensors in the healthcare sector by dealing with the biosensors for drugs, biomolecules, as well as biomarkers with smaller molecular weight, proteins, and infectious disease.

## 1. Introduction

Among key causes of mortality in different age groups throughout the world are cardio vascular and cancer diseases. Moreover, we witness the increasing growth of multiple pathogenic diseases such as viral and bacterial infections, and diseases due to exposure to toxins. Diagnosing such diseases at the initial stages would result in the use of preventative drugs instead of conventional ones [1,2,3,4,5]. Pharmaceutical drugs are used for diagnosing, preventing, and/or treating diseases through their biological effects on human bodies. Administering impurity-free and sufficient content of pharmaceutical compounds help improve the condition. Moreover, the purity and quantity of the materials in the pharmaceutical compounds must be continually checked during the drug production process using diverse instrumental or chemical analysis methods. Indeed, the possible development of impurities and changes in the chemical or quantity of active drug species at different phases such as the production, transportation, or storage, makes them redundant and dangerous for human health; therefore, medical sectors and pharmaceutical companies need precise, sensitive techniques for quantitative and qualitative analyses [6,7]. As a result of the increased misbalance in the natural metabolisms of human bodies, it is necessary to analyze diverse small biomolecules [8]. In this regard, biomedical and point-of-care uses have highly pushed the development of new diagnostic devices. Furthermore, the rapid technological progress is fueling telemedicine, electronic health (e-health), and electronic-hospital (e-hospital). Among other options, electronic health care monitoring has attracted the diagnostic and therapeutic sectors because of the increased lack of healthcare personnel [9,10]. Current progress in the bioanalytical methods has resulted in the integration of traditional biological concepts with digital instruments for establishing a simple handheld device to detect and quantify the biomedical analytes [11]. Other studies have shown that an electrochemical biosensor assay provides quantitative and qualitative determination, which has been shown to be of high importance for monitoring therapy and biomedical diagnosis of diseases that could not be obtained by traditional assays. As stated in the International Union of Pure and Applied Chemistry (IUPAC), “biosensors are a self-contained integrated tool with the capability of presenting particular quantitative or semi-quantitative analytical data with one of the biological recognition elements (biochemical receptor) that has been shown to be in direct spatial contact with the transducer element”. The analytical tools transform a chemical or biological response into an electrical signal and thus are generally categorized according to the kind of the bio-receptors engaged in biorecognition events such as enzymes, antibodies, peptides, aptamer, molecularly imprinted, and DNA. Electrochemical biosensors have been introduced to be the major analytical devices for detecting multiple biomedical analytes due to features such as self-containment, portability, robust and exact measurement, and inexpensiveness. To have an acceptable sensitivity, electrochemical biosensors must employ materials with several active sites [12,13,14,15]. MOFs have been recognized as the new molecular crystalline materials, with high attractiveness in catalysis, and biomedical uses as a result of specific structural characteristics and very good optoelectronic features. It is possible to largely enhance the function of electrochemical biosensors with the use of the MOFs as the sensing platforms because of the significant electro-chemistry features of the MOF. Moreover, MOFs are an attractive alternative as electrochemical biosensing materials due to higher mass transfer ability in the controlled porous structures [16,17,18,19]. Indeed, they carry the materials’ features and several beneficial features for electrochemical biosensor uses such as porous structures and higher specific surface areas. Such characteristics imply higher catalytic activities for target molecules, more acceptable sensitivity resulting from their major electronic features, and very good physical and chemical properties such as acceptable biocompatibility, significantly specific structural areas, and stronger interactions with biomolecules, cells, DNA, and other bio-organisms on their interfaces. Hence, MOFs could be chosen as the largely sensitive detection systems for electrochemical biosensor uses [20,21,22,23,24].

The present review discusses MOFs’ characterization and synthesis and along with some examples of their uses. The review investigates the advancements in MOFs for use in electrochemical biosensors for biomedical applications. Some challenges and/or potentials of MOFs as the electrochemical biosensors of the coming years will be provided.

## 2. Synthesis of MOFs

It is crucial to control the dimension and morphology of the MOFs’ structures. Until ten years ago, researchers used to employ several sophisticated procedures and advanced methods for synthesizing and constructing MOFs with special functions.

MOFs have been fabricated via bridging the organic linkers and metal ions or metal ion clusters. Even though it is easy to synthesize MOFs by coordinating the organic ligands and metal clusters, it is a challenge to obtain the intended structures. Furthermore, synthesis methods could influence the MOFs’ structures and features [25,26]. More recently, researchers have developed a variety of MOFs’ fabrication techniques similar to traditional synthesis (i.e., hydrothermal and solvothermal), electrochemical, mechanochemical or sono-chemical, and microwave.

### 2.1. Hydrothermal/Solvothermal Methods

Solvothermal is a more general concept than hydrothermal and refers to the application of solvents different than water. Moreover, solvothermal synthesis is performed at the solvent boiling temperature or above it into closed chemical reactors at higher pressures due to the solvent vapor or generated by a pump. Such a synthesis approach is followed by greater yields and more acceptable crystallinity of the products. Because of the greater pressure, the solvent may be heated above its boiling point (i.e., at a pressure of 1 atm), elevating the salt solubility in the reaction and increasing the reactions. Additionally, slower crystallization from a solution forms regular large crystals with a higher internal surface area. Out of the benefits of the mentioned approach, researchers have highlighted the possible total control of the synthesis conditions for a long-term process, allowing the development of reproducible protocols [27,28,29]. The hydrothermal and solvothermal approaches have been used for synthesizing several MOFs.

In this regard, Tzitzios et al. procured a nano-porous MOF, indicating an IRMOF-1 type crystalline structure, which was followed by a direct solvothermal synthesis method with the use of terephthalic acid and zinc nitrate as the precursors and dimethylformamide as the solvent in combination with the activation of super-critical CO_2_ and vacuum out-gassing techniques [30].

Moreover, Jouyandeh et al. reported the hydrothermal synthesis of MIL-101 (Cr), which is one of the MOFs with terephthalic acid as a linker and chromium as metal. To perform synthesis, researchers have applied Cr(NO_3_)_3_.9H_2_O solution and terephthalic acid in water and heated the mixture at 218 °C for 18 h [31].

### 2.2. Microwave Synthesis

Microwave-assisted chemistry provided the new ground for laboratory and industrial scales and may result in greater efficiency and productivity. The key benefits of microwave have been shown to be a shorter time of reaction, higher product yields, a greater rate of reaction, energy-efficient heating, fast optimization, very good parameter control, wider and more dynamic temperature ranges, as well as smooth article morphologies. These features make it an ideal process for future applications. Researchers have employed microwaves to synthesize MOFs due to the merits of the microwaves’ synthesis of porous substances such as faster crystallization, different morphologies and sizes, simple assessment of the reaction parameters, and phase selectivity. In addition, they synthesized MOFs using a microwave for showing faster crystallization, smaller size, and phase selectivity [32,33]. In spite of each advantage of microwave irradiation, its synthesis is seldom utilized for synthesizing the organic–inorganic material.

Furthermore, George et al. reported the fast synthesis of MIL-125 (or, Ti-BDC) MOF through microwaves. According to them, optimal synthesis time and optimal temperature equaled 40 min and 120 °C. Then, they carefully controlled variations in the development of pressure and microwave power during the synthesis condition. They observed linear development of pressure and a lower maximum value of ca. 14 bar in the course of the early heating rate time at the optimal synthesis condition. Nonetheless, according to the holding time, power and pressure were relatively fixed at nearly 10 W and 6 bar. Furthermore, the product showed to be a completely crystalline material, with the crystallite size ranging between 5 and 8 nm [34].

### 2.3. Electrochemical Synthesis

The main idea of the electrochemical synthesis of MOF has been the fact that metal ions enter not through a solution of the corresponding salt or formation of the ions during the reaction between a metal and an acid, but through an electrochemical process. In fact, metal ions enter the reaction mixture consisting of dissolved linker molecules and an electrolyte by dissolving anode, which avoids forming anions during the reaction and initiates a continual process necessary to create a lot of MOFs. To prevent the deposition of the metal cations on the cathode, protic solvents have been employed, though, hydrogen could be released within the electrochemical process. Moreover, when not used in small amounts, several solvents such as acrylonitrile, maleates, and acrylates are initially reduced [35,36].

Electrochemical synthesis has been applied to produce Cu_3_(BTC)_2_ MOF, which has been utilized as a catalyst to reduce the chemical of nitrophenol in the presence of excessive NaBH_4_. An optimized condition for electrochemical synthesis of Cu_3_(BTC)_2_ via anodic dissolution of copper ion for binding with the BTC molecules (organic linker) was demonstrated using a constant voltage between copper plates working as the anode and the cathode with tetrabutyl ammonium tetrafluoro borate (TBATFB) as the supporting electrolyte. Merits of this synthetic procedure provided greater yield, higher surface areas, and purity than the conventional processes [37].

Moreover, Wei et al. addressed the synthesis of a Zr-based ultra-stable MOF, UiO-66-NH_2_ using an electrochemical approach with the use of metal Zr as the metal source under atmospheric pressure and room temperature [38].

### 2.4. Mechanochemical Synthesis

In general, the mechanochemical method entails the completion of the chemical reactions with the assistance of mechanical force. Such a method is used for fast reactions without or with a small content of solvent and yields a small volume of product [39,40].

In their research, Chen et al. have shown a fast effective path of mechanochemical synthesis of InOF-1 with a large surface area. Then, they optimized the required conditions for the mechanochemical synthesis of InOF-1: powdering In(OAc)_3_·6H_2_O and H4bptc as the starting material with the addition of 0.4 mL CH_3_CN for 20 min. Interestingly, InOF-1 exhibited exceptional water stability and it was demonstrated the use of the liquid-assisted grinding for 20 min with CH_3_CN (0.4 mL) may result in the largely porous, crystalline InOF-1 with a surface area of 707 m^2^/g [41].

Chen et al. employed a liquid-assisted mechanochemical approach for synthesizing the copper-based MOF-505. According to them, the synthesis parameter of the assisted solvent (content and size) greatly influenced the porosity and crystallinity of the mechanochemical reaction product in comparison to the grinding time. After adjustment of the parameters, grinding H4bptc and Cu(OAc)_2_·H_2_O with 0.4 mL DMF assisted for 80 min achieved MOF-505-K with the greatest surface area that equaled 977 m^2^/g [42].

### 2.5. Sonochemical Synthesis

Sonochemistry refers to acoustic cavitation, formation, growth, as well as the implosive collapse of the bubbles in a liquid. In fact, the collapse of the bubbles creates localized hotspots with a temporal temperature of nearly 5000 K and a cooling rate >10^10^ K/s. Moreover, decomposition of the volatile organometallic compounds in the cavitating bubble would occur for yielding metal atoms that agglomerate to produce diverse largely porous nano-structured substances. In addition, sonochemical approaches may result in homogeneous nucleation and a higher decline in the crystallization period than that of the previous oven heating in the preparation process of the materials [43,44].

This approach gave Cu_3_(BTC)_2_ (HKUST-1) from a solution of H_3_BTC and an aqueous solution of copper acetate in a mixture of DMF with ethanol. The starting compounds were put in a container mounted in a water bath and exposed to sonication at a frequency of 40 kHz for some time between 5 and 60 min. According to the analysis, the product yield ranged between 62.6% and 85.1%, depending on the synthesis time [45].

Furthermore, Cho et al. addressed the preparation of ZIF-8 with the use of a sonochemical technique based on the pH-adjusted synthesis condition with NaOH (aq) using few triethylamine. Following the optimization of the synthesis factors, the method has been extended to 1 L-scale synthesis with the yield of the product of 85% at the higher substrate concentration (Zn^2+^:DMF = 1:9.3) within 2 h without any damages to the textural features [46].

## 3. Characterization of MOFs

A variety of physicochemical methods are needed to detect the characteristics of MOFs. The fundamental characterization consists of nitrogen (N_2_) adsorption/desorption isotherms for confirming porosity and calculating the surface area along with powder X-ray diffraction (PXRD) pattern for establishing phase purity and crystallinity of the materials. Other approaches of MOF’s characterization are: (1) scanning electron microscopy (SEM) for measuring the crystals’ size and morphology that may be coupled with the energy-dispersive X-ray spectroscopy (EDS) to obtain considerable data on the distribution and elemental composition, and transmission electron microscopy (TEM) for determining the size of the grains and particles; (2) thermo-gravimetric analysis (TGA) for determining the thermal stability of the MOFs and, in several situations, for estimating the volume of the pores; (3) Fourier-transform infrared spectroscopy (FT-IR) that may be utilized for confirming the presence (or absence) of the IR active functional groups in the framework; (4) nuclear magnetic resonance (NMR) spectroscopy that may be employed for determining the bulk purity of a specimen and the quantification of the linker ratio in the mixed linker MOFs. Here, all approaches to characterize MOFs will be illustrated.

### 3.1. N_2_ Adsorption/Desorption Isotherms

It is possible to use adsorption isotherms for non-reactive gases under cryogenic temperature to determine the surface area for MOFs, their pore volume, and distribution of the pore size. The above method addresses N_2_ adsorption onto the solid surface at the boiling temperature of liquid nitrogen (77 K), which causes an adsorption isotherm. Finally, the isotherm shape presents data on the solid homogeneity [47].

The IUPAC, in 1985, presented a classification of six kinds of physisorption isotherms and introduced the classification of the pores by the width of the internal pore [48].

Moreover, Thommes et al.’s study dealt with the refinement of the original IUPAC classification of the physisorption isotherms, wherein Type-I and Type-IV isotherm have been grouped into I(a), I(b), IV(a), and IV(b). This condition occurs with the kinds of hysteresis loops. Recent studies have obtained two other types, leading to six kinds of hysteresis loops associated with the pore structure and basic absorption mechanism [49]. Generally, MOFs have been proposed to be solids with a higher surface area, that is, greater than 2000 m^2^·g^−1^, and porosity so that most micro-porous substances characterize the type-I isotherms. Now, the optimal default approach would be the application of the Brunauer–Emmett–Teller (BET) theory for computing the surface area due to the supports of the multi-layer gas absorption from the pore size of most of the MOFs [50].

BET area is assessed in two phases. Firstly, a physisorption isotherm is transformed into a ‘BET plot’, then the value of the BET mono-layer capacity in nm is derived. The second phase involves the specific surface area (S) that demands information about the molecular cross-sectional area. Absorption isotherm with the BET equation allows to calculate of the monolayer capacity n_m_:

1/[n((P_0_/P) − 1)] = 1/(n_m_C) + [(C − 1)/(n_m_C)] (P/P_0_) (1)

where n represents the absorbed content, n_m_ refers to the mono-layer capacity, and finally C implies an empirical constant, which indicates the magnitude order of the adsorbent-adsorbate interaction; moreover, P stands for pressure and P_0_ represents the saturation pressure of N_2_. It is necessary to choose a linear area with regard to the criteria introduced in Rouquerol et al.s’ study, demonstrating that (1) the BET constant “C” should be positive, (2) n(1 − P/P_0_) must enhance with P/P_0_ monotonically, (3) the monolayer capacity (n_m_) must correspond to a pressure in the limits of the data, and (4) The computed value for monolayer formation (1/(√C + 1)) must be nearly equal to P/P_0_ at the monolayer capacity. It is possible to extract the intercept and slope of the linear area by BET area (apparent surface area), n_m_ [51,52,53,54].

In addition, Sargazi et al.s’ study dealt with the synthesis of a Ni-MOF with the use of ultrasound irradiation. Researchers employed N_2_ absorption approaches (measured at 77 K) to determine the pore textural features such as surface area (2021 m^2^/g), the diameter of the pores (11.7 Å), as well as the volume of the pores (0.882 cm^3^/g) of the ultrasonic, assisted synthesized Ni-MOF at optimal conditions. Notably, textural features of the MOFs used in their study differed from earlier investigations on the surface areas and pore volumes. This issue may be caused by ultrasonication on the textural features of the resultant product (ultrasonic impact) [55].

### 3.2. PXRD

PXRD is one of the commonest approaches to analyze the structures and crystallinity of MOFs. In the case of the crystallinity of a sample, it is possible to extract further data from the powder pattern, including the unit size of the cell. Researchers compared experimental powder patterns with the simulated patterns produced through single-crystal X-ray data or using computational modeling. Current advanced approaches to analyze PXRD data have been designed in a way that it is possible to regularly determine the crystalline structures, differentiation between crystalline and amorphous materials, various polymorphic forms, as well as percent of crystallinity of the substances from PXRD data [56,57,58].

In their study, Butova et al. described the synthesis and the relative characterization of a set of UiO-66 MOFs with 1,4-benzenedicarboxylate (C_6_H_4_(COOH)_2_, referred to as H_2_BDC) and 1,4-naphthalenedicarboxylate (C_10_H_6_(COOH)_2_, referred to as H_2_NDC) mixed linkers with the NDC volume of 0, 25, 50, and 100%. Initially, they used a laboratory-level PXRD D2 Phaser system (Bruker) operating with Cu Kα radiation (λ = 1.5418 Å) to determine the phase purity of the UiO samples. Based on the outputs obtained from laboratory PXRD characterization of the samples, an acceptable level of crystallinity, as well as phase purity of these samples, have been observed that verified the UiO-66-like phase in each case. Synchrotron PXRD data in the 2θ range of 4.5–10.5° provided comprehensive structural analysis [59].

### 3.3. SEM and TEM

SEM has been considered as one of the beneficial tools for measuring diverse features of MOFs. This tool produces high-resolution two-dimensional (2D) images, displaying the shape of materials and their spatial changes, which reflect data on the exterior morphologies, the mixture of phases, and dispersion; however, the porous structures characterizing the MOFs offer particles of attractive forms such as bars, cubes, and rhombohedra, which yield a different morphology. Because of the insulating nature of many MOFs, image artifacts such as the charging effect may frequently experience detraction, or in several situations, complete obstruction of achievement of the high-quality SEM images. Hence, coating the specimens with conductive materials such as platinum or gold would be crucial for decreasing the charge buildup from the electron gun [60,61].

For resolving the mentioned shortcoming, experts in the field can use field emission scanning electron microscope (FESEM) that has been devised according to technology for high-resolution imaging and various approaches, which aim at the complete characterization of the samples. In fact, FESEM employs the field emission gun as an emitter type that presents the highly focused electron beams, enhancing the spatial resolution working at a lower potential; therefore, it is possible to commonly use this tool for imaging surface sensitive and nonconductive specimens without any necessity for pre-treatment [62]. Put differently, TEM has been introduced as one of the strong tools for determining the size of the grains, size of the particles, as well as crystallographic information such as the plane dislocation and index. Moreover, it is possible to employ a variety of software for analyzing the microscopy images such as Cell Profiler and Image J. TEM has shown its usefulness, particularly in the characterization of MOFs modified by incorporation of nanoparticles (NPs) due to accessibility of data on the NPs’ dispersion and dimension [63,64]. EDS and SEM reflect the semi-quantitative and qualitative results and can present basic data of the material composition of the scanned specimen that cannot be offered by the previous laboratory experiments [60].

**Figure 1:**
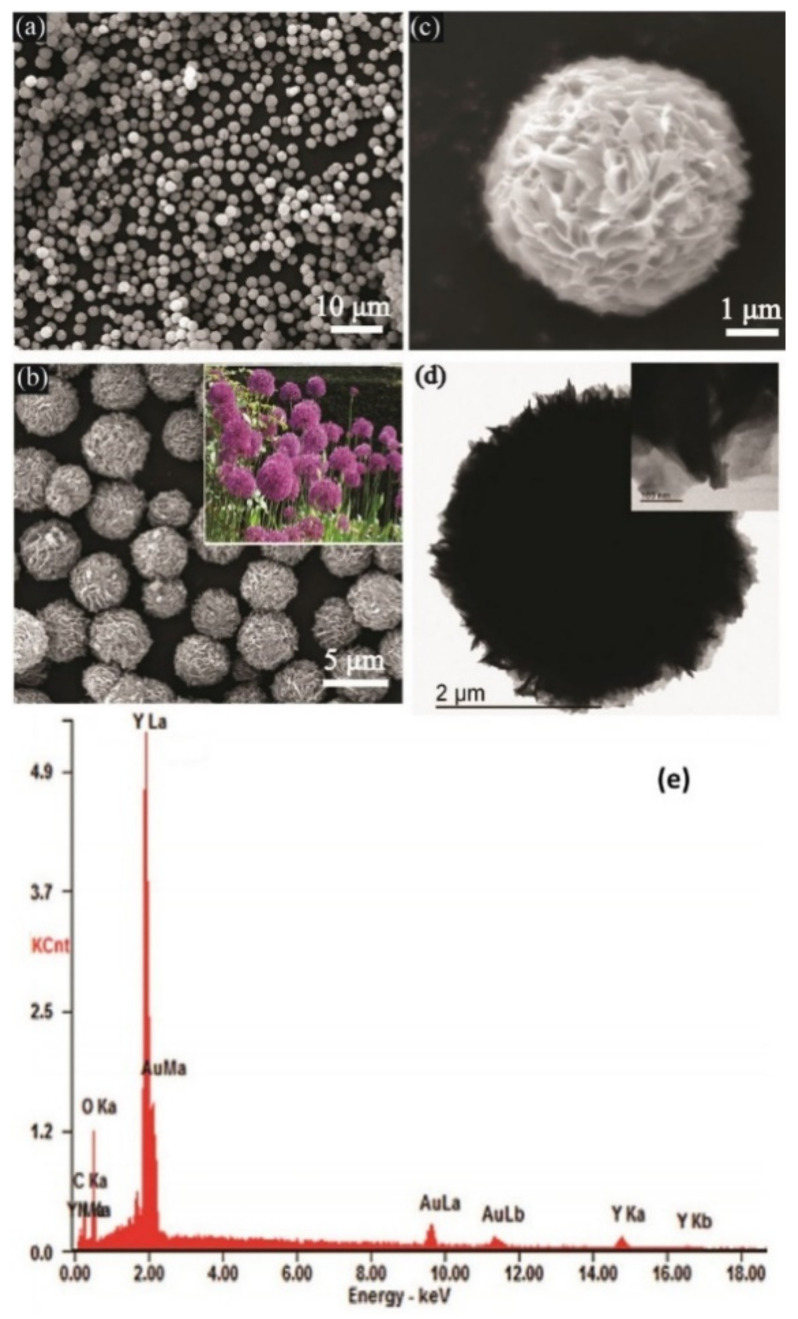
TEM and SEM images of the as-prepared samples of Y-MOFs at 120 °C for 24 h: (**a**–**c**) low-magnification and high-magnification images obtained from SEM (inset in (**b**) relates to allium giganteum); (**d**) low-magnification and high-magnification images obtained from TEM; (**e**) EDX spectra. Reprinted with permission from Ref. [65].

Yang et al. presented the synthesis of yttrium-based MOFs (Y-MOFs) through TEM, EDS, and SEM investigation. They used SEM for examining the morphology as well as micro-structural characteristics of the as-prepared Y-MOFs samples. Figure 1a depicts the related pattern. As shown in the figure, the samples exhibited a smooth dispersion of the sphere-like architecture with sizes from 3 to 5 μm. Figure 1b,c show the expanded SEM images. The whole structure of the spheres contains numerous nanosheets with nearly 200 nm width and 40–50 nm thickness. The image looks like a potted plant known as allium giganteum (inset in Figure 1b), which has named the samples’ morphology as allium giganteum-like. Regarding Figure 1d, the low-magnification and high-magnification images obtained by TEM revealed the assembly of the nanosheets in a radial form from the center to the surface. Further, EDX spectra of the samples in Figure 1e reveal the presence of C, Y, O, and N elements consistently with the element composition of the target samples of Y-MOFs [65].

### 3.4. Thermogravimetric Analyses

Thermogravimetric analysis has been proposed as one of the direct methods to investigate the MOFs’ thermal stability. It measures the mass of the examined sample as a function of temperature. It is possible to use the TGA spectra of a solvated specimen to estimate the volume of the pores. Decomposition of MOF changes according to the carrier gas (i.e., air, O_2_, and N_2_) that is employed for TGA. It is notable that a thermo-gravimetric analyzer would be frequently combined with a mass spectrometer to determine not only the temperature for changing the samples’ mass but also the molecules involved in the mass changes [66].

Ref. [67] reports a TGA curve with MOF weight loss (%) versus temperature with the first loss of mass in ranges from 100 to 150 °C that relates to the removal of unreacted ligands as well as to the guest water molecules in the pores. Moreover, decomposition temperature is observed at ~460 °C in N_2_ for ensuring the higher thermal stability of MOF; however, weight loss would not result from the structural variations; therefore, the research must be complete with XRD analyses at different temperatures for checking the variations of the structures [67].

### 3.5. Fourier Transform Infrared Spectroscopy

It is widely accepted that it is possible to use FT-IR for probing the absence or presence of the IR active functional groups in an MOF. Upon the availability of a specific sample holder, we can obtain more complicated data such as the MOFs’ behavior in the presence of gases such as CO_2_ and CO or at different temperatures. Moreover, a simple operation of FT-IR could be obtained on the powdered specimens of an MOF, or dilution of the specimens could be obtained in matrix-like potassium bromide (KBr). A method for the samples’ dilution is the physical mixing of the MOFs with KBr with a pestle and mortar. Due to the instability of several MOFs against mechanical stress, the sample must be ground carefully. According to the suggested approach, infra-red radiation from an infrared source is used for irradiating the samples, and thus vibrational motions are stimulated by absorbing this radiation via deposition of quanta of energy into the vibrational states. Hence, upon the exposure of a molecule to the radiations generated by thermal emission of a hot source (e.g., IR energy), it is absorbed merely at the frequencies relative to its molecular mode of vibration in an area of electromagnetic spectra between the short waves (micro-waves) and visible (red); therefore, variations in the vibrational motion result in the bands in the vibrational spectra so that all of them would be characterized by amplitude and its frequency [68,69].

In their study, Zhao et al. provided the synthesis of one of the new gadolinium-porphyrin MOF nanosheets via coordinating chelation between 4,4,4,4-(porphine-5,10,15,20-tetrayl) tetrakis (benzoic acid) (TCPP) and gadolinium(III) ions (Gd). Then, they examined the creation of Gd-TCPP and used FT-IR to characterize it. Researchers attributed the adsorption peak at 963 cm^−1^ and 3315 cm^−1^ to the N-H stretching vibration and N-H in-plane vibrations. The adsorption peaks have been constant following the creation of the Gd-TCPP nanosheets, which reflects the inability of the hydrogen proton to be replaced by the gadolinium ion. Results have also shown the significant weakening of the C=O stretching vibration at 1683 cm^−1^ from the carboxyl functional groups when the coordination band formed, and a new absorption peak has been observed at 1651 cm^−1^, extracted from the C=C stretching vibration of porphyrin. This current absorption peak at 1582 cm^−1^ could be assigned to the general skeleton vibration created by the MOF nanosheet structure. Moreover, the absorption peak at 1485 cm^−1^ could be derived from the sulfonic acid groups that have been weakened following the creation of the coordination bonds. A similar adsorption peak has been observed at 1653 cm^−1^ for Gd-TPPS because of the C=C stretching vibration, which verified the same impact of the production of the coordination bond on the infrared spectra of both porphyrin molecules [70].

### 3.6. NMR Spectroscopy

It is possible to use this method to determine the MOFs’ purity, leftover modulator, the absence of solvent after activation, and linker ratio. Since a lot of MOFs cannot be solved in the earlier NMR solvents, we need MOF digestion before obtaining a spectrum. The commonest approach to the MOF digestion for NMR purposes requires the addition of 5 to 10 drops of D_2_SO_4_ to 1–2 mg of MOF and sonication of the mixture until a complete dispersion of the sample in acid. In the next step, we could add ~0.5 mL of DMSO-d_6_ for dilution and full dissolution of the mixture (maybe heating and sonication are needed following the addition of DMSO-d_6_ for dissolving the sample) [71].

In their study, Klein et al. examined the synthesis and characterization of one of the novel members of flexible MOFs called Ni_2_(2,6-ndc)_2_(dabco (DMF)_6.5_(MeOH)_3_ (DUT 8(Ni)). It showed reversible crystal-to-crystal transformation when the solvent had been removed and many gases had been absorbed. Analyses of xenon adsorption along with the Xe NMR spectroscopy are also a desirable approach to determine and characterize the so-called gate-pressure effect in the proposed MOF material. Moreover, researchers demonstrated that chemical shift and line width of the Xe NMR signal would be sensitive variables to determine the structural transition from a narrower pore system of lower porosity to a wider pore system. Finally, clear detection of threshold temperature and the transition has been observed [72].

## 4. MOF-Based Electrochemical Biosensors for Biomedical Uses

### 4.1. Detection of the Pharmaceutical Drugs

Therapeutic drugs contribute importantly to human life and are used increasingly so that USD 1.52 trillion would be spent on the pharmaceutical sector by 2023. This fast trend is caused by the global aging population as well as the appearance of new pandemics and diseases such as COVID-19; therefore, monitoring of therapeutic drugs has been proposed for managing treatments in the infected cases, in particular, for the medicines with the narrow therapeutic range. In fact, therapeutic ranges refer to the differences between the concentration of a drug with minimal effect and the concentration of that drug with minimal toxicity. Nonetheless, pharmaceutical analyses must be conducted during each step of the pharmaceutical preparation, that is, from the initial phase of synthesis, stability tests, and quality control to the pharmacological and toxicological experiments in animals and humans. Following administration to the infected cases, analytical measurements would be crucial to evaluate their efficiency and examine the favorable dose of the drug formulation [73,74,75,76]. Moreover, it is necessary to perform quantitative analyses of the active ingredient volume in the drugs’ formulation and follow the administration in the human biological fluids.

MOF-based electrochemical biosensors are designed to determine the pharmaceutical drugs as listed in Table 1.

Hu et al. designed an effective, sensitive, and simplified electrochemical immuno-sensor based on the Au NPs/Zn/Ni-ZIF-8-800@graphene composites for monensin detection in milk. Then, researchers obtained Zn/Ni-ZIF-8-800 with higher stability through pyrolysis of the Zn/Ni-bimetallic MOFs precursor Zn/Ni-ZIF-8. Using the synergistic effect such as higher porosity of Zn/Ni-ZIF-8-800 as well as the very good electrical conductivity of graphene, the electrochemical signal is largely elevated. In the next step, Au NPs have been electro-deposited on the surface of Zn/Ni-ZIF-8-800@graphene to capture antibody-antigen. Results showed a higher specific surface area of these new hybrid substances and very good capacity for electron transfer and biocompatibility. Cyclic voltammetry (CV) has also been used to examine the development procedure of the biosensor and differential pulse voltammetry (DPV) has been employed for the determination process. At ideal conditions, the linear ranges equaled 0.25 and 100 ng mL^−1^, and limit of detection (LOD) has been 0.11 ng mL^−1^ [77].

**Figure 2:**
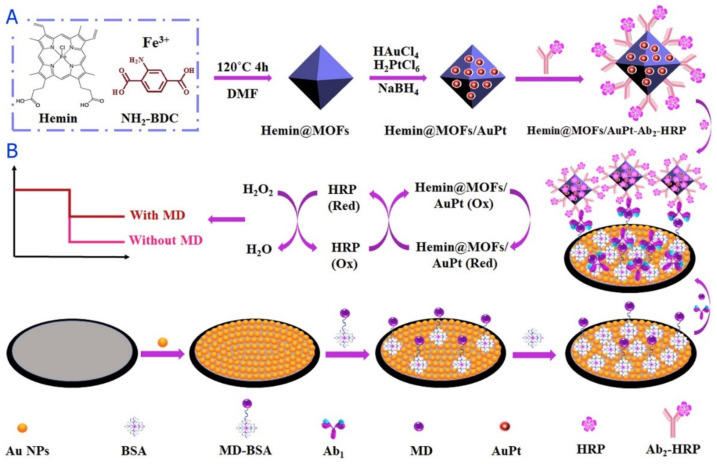
(**A**) Development steps of the hemin@MOFs/AuPt-Ab2-HRP/HRP bioprobes; (**B**) the suggested electrochemical immunosensor to detect maduramicin and the respective signal amplification method. Reprinted with permission from Ref. [78].

As mentioned in several studies, maduramicin (MD) has been introduced as one of the types of mono-glycoside polyether ionophore antibiotics with the effects on the treatment of coccidiosis and facilitation of the growth of animals. Nevertheless, excess and widespread utilization of this antibiotic causes potent risks to human health; therefore, Hu et al.s’ study addressed the fabrication of an electrochemical immunosensor according to the indirect competitive protocol to analyze the MD residues in eggs by a multi-signal amplification device. Researchers observed deposition of the Au NPs on the surface of the glassy carbon electrode (GCE) for loading the coating antigen MD-BSA and improving conductivity. In the next step, they encapsulated hemin into Fe-MIL-88 NH_2_ (hemin) MOFs by constructing the signal amplification platform and used AuPt NPs for decorating these new composites. In fact, the synthesized hemin@MOFs/AuPt has been employed as a signal amplification mediator and as a carrier to immobilize the HRP and HRP-conjugated affinipure goat anti-mouse antibody (Ab_2_-HRP). Moreover, these hemin@MOFs/AuPt-Ab_2_-HRP bioconjugates may highly amplify the current signal because AuPt, HRP and hemin@MOFs had higher catalytic activities toward hydrogen peroxide. Figure 2 represents the development process of hemin@MOFs/AuPt-Ab2-HRP/HRP signal bioprobes as well as the sensing mechanism of this new immunosensor. This immunosensor exhibited higher stability and sensitivity during detection. Finally, the synergistic catalytic effect of hemin@MOFs, AuPt, and HRP has been followed by the wider determination ranges between 0.1 and 50 ng mL^−1^ and a lower LOD of 0.045 ng mL^−1^ (S/N = 3) [78].

It has been found that the stereochemical configuration of the drugs contributes to the racemic switch with enantiomers in the presence of a chiral environment for humans. Hence, researchers face challenges in its detection in pharmaceutical and racemic samples. For this reason, Upadhyay et al. used square wave voltammetry (SWV) and presented an enantio-selective electrochemical biomimetic sensor to discriminate isomers of ethambutol (ETB); therefore, they synthesized a chiral host, β-Cyclodextrin-based copper (CD-Cu) MOF, and employed it for chelate complexation of the ETB isomers (SS-ETB/RR-ETB). Researchers observed the chemical modification of a GCE with the composite materials of carbon nanofibers (CNF) and CD-CuMOF for constructing a sensor as CD-CuMOF-CNF-GCE. Researchers assumed that one of the artificial enzyme models (AEMs) is the behavior of CD-CuMOF for ETB isomers on the GCE because of the imitation of the catalytic activities that are similar to enzyme alcohol dehydrogenase for ETB. This new biosensor exhibited a very good peak potential difference (ΔEp (SS − RR) = 108 mV) between the ETB isomers with the use of SWV that indicated an obvious difference in the racemic mixture. Additionally, it linearly responded in ranges from 1.0 × 10^−7^ to 1 × 10^−4^ M and 5.0 × 10^−7^ to 2.5 × 10^−4^ M with lower LOD of 8.52 × 10^−8^ M and 3.10 × 10^−8^ M for SS-ETB and RR-ETB isomers. Finally, this sensor has been employed to estimate the ETB isomers in the real samples such as drug, blood, and urine as well as racemic mixture [79].

In another study conducted by Wu et al., an electrochemical biosensor has been fabricated according to a water-stable 1D double-chain Cu(II) MOF (Cu-MOF) for effective recognition of L-tyrosine (L-Tyr) under biomimetic conditions. Wu and coworkers used a hydrothermal approach for synthesizing the Cu-MOF: {[Cu(bpe)(fdc) (H_2_O)(DMF)]·0.5H_2_O}n (bpe = 1,2-di(4-pyridyl)ethylene, H_2_fdc = 2,5-furandicarboxylic acid; i.e., Cu-1). They coated it on the surface of GCE for preparing an electrochemical biosensor (Cu-1/GCE) with better biosensing abilities towards L-Tyr. The suggested Cu-MOF electrochemical biosensor exhibited higher sensitivity and facile functioning towards L-Tyr in the concentration ranges between 0.01 and 0.09 mM. Finally, LOD equaled 5.822 μM and Cu-1/GCE displayed very good selectivity towards L-Tyr under biomimetic conditions with numerous amino acid interferents [80].

In another study conducted by Song et al., an electrochemical biosensor has been fabricated based on a bimetallic 2D semiconducting MOF materials for detection of enrofloxacin. Song and coworkers synthesized CoNi-based MOF, CoxNi_3-x_(HITP)_2_, by using 2,3,6,7,10,11 hexaaminotriphenylene (HATP) as an organic linker. The suggested CoxNi_3-x_(HITP)_2_-based electrochemical biosensor exhibited higher sensitivity and facile functioning towards enrofloxacin in the concentration ranges between 0.001 pg·mL^−1^ and 1.0 pg·mL^−1^. Finally, LOD equaled 0.2 fg·mL^−1^ as well as CoxNi_3-x_(HITP)_2_-based biosensor displayed very good selectivity, good reproducibility, high stability, and wide applicability [81].

Zhang et al. developed a strategy to fabrication C_60_@UiO-66-NH_2_ nanocomposites by combining isolated fullerenes (C_60_) molecules into the inner pores of nano-scale MOFs. MOF nanoparticles provide more Zr(IV) sites to coordinate with phosphate groups for increased immobilization of aptamers and enhanced detection signals; however, electro-active C_60_ with a small size can be incorporated into porous skeletons to obtain a nano-scale hybrid for improving the electrical activity of pristine MOFs. C_60_@UiO-66-NH_2_ nanocomposites serve as excellently promising materials to modify electrodes and stabilize aptamers, resulting in a significant biosensor for impedimetric biosensing of tobramycin (TOB). At optimum condition, this aptasensor displayed the linear concentration ranges between 2.1 × 10^−3^ nM and 106.9 nM, with a lower LOD of 0.377 pM. It could be concluded that their aptasensor had satisfactory selectivity, stability, and reproducibility [82].

### 4.2. Detection of Disease Biomarker

As described by the National Institutes of Health, biomarkers have been introduced as compounds, which can be assessed and measured objectively as one of the indicators of normal biological and pathogenic processes, or pharmacological response to treatment intervention. Biomarkers can be localized in the tissues, blood, and urine [83,84]. Moreover, changes, presence, and absence of the biomarker concentration reflect if a person is threatened by a disease if there is an earlier disease in the body, and how the disease can spread. Physicians, epidemiologists, and scientists employ biomarkers to examine disease, and thus the precise determination of biomarkers would prevent diseases, accompanied by more satisfactory results for the health of the patients, and largely decline the health care expenses [85,86]; therefore, it has been tried to overview the current studies on the major biomarkers associated with the most critical diseases. Here, we report the major review papers on the chosen biomarkers. Table 2 is a summary of several investigations, which addressed the fabrication of electrochemical biosensors to determine disease biomarkers.

**Figure 3:**
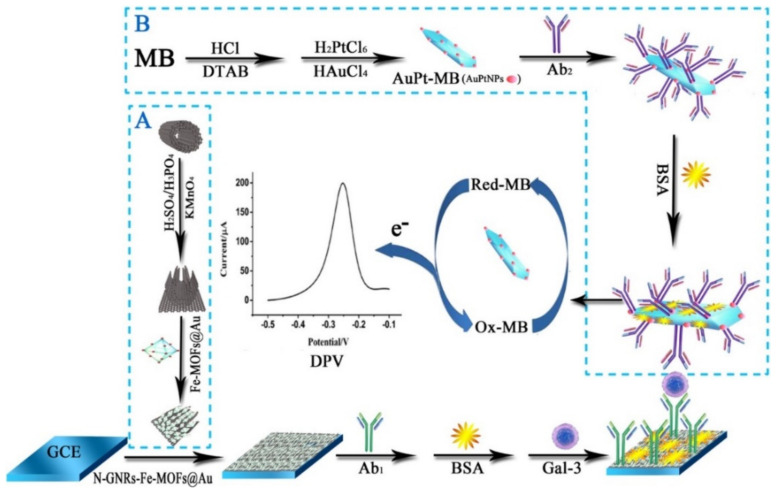
Assembly of the interface of electrochemical immunosensor. Preparation of (**A**) N-GNRs-Fe-MOFs@Au NPs and (**B**) AuPt-MB-Ab_2_ bioconjugate. Reprinted with permission from Ref. [87].

Moreover, Tang et al. devised an electrochemical immunosensor to detect Galectin-3 (Gal-3), which is one of the biomarkers of heart failure. They modified GCE with a film of a composite constructed from the N-doped graphene nano-ribbons immobilized Fe-based-MOFs, coated with Au NPs (NGNRs-Fe-MOFs@AuNPs). Then, the primary antibody against Gal-3 (Gal-3-Ab1) was immobilized on the Au NPs over the surface of the modified GCE that was obstructed by bovine serum albumin (BSA) (see Figure 3A). For the first time, researchers synthesized one kind of novel redox-active species, AuPt Methylene blue (MB) (AuPt-MB) nano-composites using a one-pot approach (Figure 3B). In fact, MB is one type of electron transfer mediator in an amperometric biosensor that contributes to the creation of electrons and amplification of the signals. According to the analysis, the rod-like AuPt-MB nano-hybrids had smooth morphologies and acceptable electrochemical activities and thus can combine with the second antibody against Gal-3 (Gal-3-Ab_2_). Afterwards, AuPt-MB-Ab_2_ coupled with the N-GNRs-Fe-MOFs@AuNPs-Ab_1_ have been used for forming a sandwich-type configuration, which may largely elevate the sensitivity of the biosensor. At optimum condition, this new immuno-sensor displayed the linear concentration ranges between 100 fg mL^−1^ and 50 ng mL^−1^, with a lower LOD of 33.33 fg mL^−1^ (S/N = 3) for Gal-3 in the spiked sera. It could be concluded that their immunosensor had satisfactory selectivity, stability, and reproducibility [87].

Additionally, Li et al. showed the synthesis of a new kind of multi-functional iron-based MOFs (PdNPs@Fe-MOFs) through the assembly of the palladium NPs on the surface of the Fe MIL-88NH_2_ MOFs micro-crystals. They utilized it in the electrochemical biosensor to detect microRNA-122 ultrasensitively (miR-122 is one of the biomarkers of drug-induced liver injuries). In fact, nanohybrid has been employed as the optimum nano-carriers to immobilize the signal probes and as the electro-catalysts and redox probes. Researchers constructed two hairpin probes as the signal and capture probes. Then, they utilized the nano-hybrids conjugated with biotinylated and signal probes and streptavidin as the tracer labels, and sandwiched the target miR-122 between the thiol-terminated capture probes and tracer label embedded in the MCH monolayer over the Au NPs functionalized nitrogen-doped graphene sheets (AuNPs@N-G) modified electrode. According to the target catalyzed hairpin assembly, it is possible for the target miR-122 to stimulate the hybridization of signal probes and the capture probes for its release for initiation of another reaction process that led to multiple tracer indices anchored on the sensing interface. As a result of the innate peroxidase-like activities of the nano-hybrids and in the presence of H_2_O_2_, it is possible to largely elevate the detection signal for electro-catalytic oxidation of 3,3′,5,5′- tetra methyl benzidine. Finally, researchers observed the wider determination ranges between 0.01 fM to 10 pM with the lower LOD of 0.003 fM (S/N = 3) using the PdNPs@Fe MOFs mimetic coreaction to amplify the signals and assemble the target catalyzed hairpin [88].

Furthermore, Xu et al.s’ study addressed the synthesis of the hollow nano-box (HN) MOFs’ nanocomposite and employed it in a signal that declined electrochemical immunosensor to detect lymphocyte activation gene-3 (LAG-3) biomarker ultra-sensitively quantitatively. Using the signal materials such as biotin-streptavidin system and SiO_2_-tagged anti-LAG-3 antibody (SiO_2_-Ab_2_), this sensor could amplify the signals. Then, researchers encapsulated the tin dioxide (SnO_2)_-functionalized rGO and Au and platinum alloys over the surface of the HN-MOFs for preparing the rGO-SnO_2/_hollow nanobox-MOFs/AuPt alloys (rGO-SnO_2_/HNMs/AuPt) as the matrix. In fact, SiO_2_-Ab_2,_ which has been applied as the signal-decreased label, could be employed for enhancing the differentiation of the electrochemical signal following the special determination between antigens and antibodies because of larger steric hindrance. At optimum conditions, the sandwich-prepared immuno-sensor sensitively detected the LAG-3 protein in the concentration ranges between 0.01 ng·mL^−1^ and 1 μg·mL^−1^, with the LOD equal to 1.1 pg·mL^−1^ (3σ) [89].

In another study, Zhang et al. reported one of the novel signal amplification approaches via coupling of the cascade catalysis-initiated radical polymerization with the impedimetric immunoassay to detect carbohydrate antigen 15-3 (CA15-3) ultra-sensitively; therefore, researchers utilized the copper (Cu)-based MOF NPs, as the peroxidase mimics in combination with glucose oxidase (GOx) and CA15-3 antibody (Ab_2_) as immuno-probes for initiating the radical polymerization through cascade catalysis. They used GOx for catalyzing glucose oxidation for forming H_2_O_2_ that reacts with acetylacetone (ACAC) by catalyzing the Cu-MOF for yielding ACAC radicals to polymerize N-isopropylacrylamide (NIPAM). Then, they produced the polymer poly (N-isopropylacrylamide) (PNIPAM) in situ from the radical polymerization. PNIPAM, as the resistance enhancer, has been covered on the surface of the electrode for amplifying the resistance value by its weak conductivity. Researchers also used polymerization-based amplification for significant improvements in the resistance difference due to the target. At the optimal condition, this new biosensor exhibited wider detection ranges between 10.0 μU/mL and 10.0 mU/mL as well as 10.0 mU/mL and 100.0 U/mL, with ultra-low LOD of 5.06 μU/mL for CA15-3 [90].

**Figure 4:**
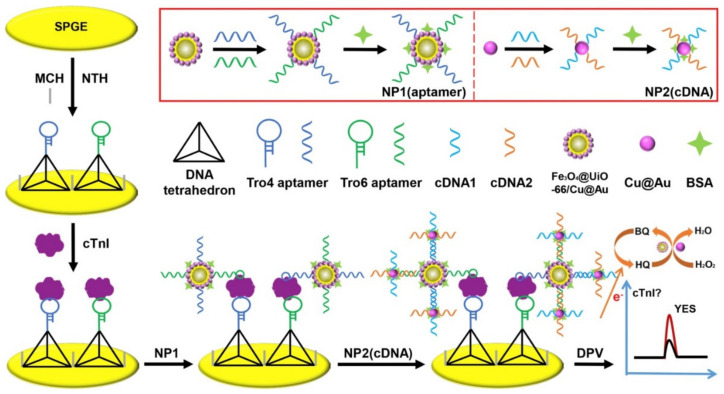
Assembly process of the non-enzymatic nano-probes and electrochemical aptasensor for cTnI capture and determination. Reprinted with permission from Ref. [91].

Sun et al. designed an electrochemical biosensor with the use of the mMOF nano-catalysts and DNA nano-tetrahedron (NTH) based on the dual-aptamer probes for nonenzymatic determination of cardiac troponin I (cTnI) that is one of the best standard biomarkers for initial diagnosis of AMI. Figure 4 depicts the development principle of the non-enzymatic electrochemical aptasensor. They immobilized the NTH-assisted dual-aptamer (Tro6 and Tro4) capture probes over the SPGE for the largely enhanced capture of the target cTnI with constant support and optimal interface density. In the next step, bimetallic Cu@Au NPs and both aptamers have been employed for decorating the mMOF Fe_3_O_4_@UiO-66 nanozymes; therefore, researchers could use this new non-enzymatic nano-probe1 (NP1) to determine the cTnI and amplify the current signal via catalysis of hydroquinone (HQ) oxidation to benzoquinone (BQ) with H_2_O_2_. In the next step, the target proteins have been obtained for fabricating a super-sandwich-like structure over the interface of an SPGE. Anchoring of the nano-probe2 (NP2) of the Cu@Au nanozymes labeled with dual-complementary DNA (cDNA) to the dual-aptamer has been observed on NP1 by hybridizing DNA, which formed the cluster-based nano-probes to elevate the sensitivity of detection. It has been found that the enzyme-free electrochemical aptasensor had a significant analytical function with dynamic ranges between 0.05 and 100.0 ng/mL, a lower LOD of 16.0 pg/mL, acceptable repeatability, and higher selectivity [91].

Chen et al. focused on the development of one of the simplified ways for the synthesis of biocompatible invertase enzyme-modified MOF materials and addressed its utilization in the construction of dual-response Dam DNA methyltransferase (MTase) sensors; therefore, they employed such sensors as the signal probes, because they can detect the higher density of metal sites electrochemically and invertase can hydrolyze sucrose into glucose to obtain the glucometer signal output. Dual-response for precise determination of Dam MTase was achieved. Chen et al. observed the occurrence of methylation of hairpin probe 1 (HP1) in the presence of Dam MTase, which cleaved HP1 helped by endonuclease (DpnI) for the production of the binding sequences. In the next step, the binding sequences were hybridized with the electrode assembled HP2 for exposure to the sticky termini that was followed by the sequential hybridization with the Invertase/MOFs-tethered capture probes. In the end, researchers showed the electrodes’ incubation with a sucrose solution for electrochemical detection and as a glucometer. This assay exhibited acceptable functions with the ability to detect Dam MTase activities of 0.001 U mL^−1^ with wider linear ranges and acceptable selectivity against other cytosine MTase (M.SssI MTase) [92].

Luo et al. designed an ultra-sensitive voltammetric aptasensor for analyzing cardiac troponin I (cTnI). This aptasensor was based on the DNA nano-tetrahedron (NTH) linked dual-aptamer (dAPT) and magnetic MOFs of Fe_3_O_4_@UiO-66 type; therefore, they immobilized the DNA NTH-linked dAPT (Tro6 & Tro4) over a Au electrode to enhance the capture effect of cTnI. Au@Pt NPs, G-quadruplex/hemin (GQH) DNAzyme, horseradish peroxidase (HRP), as well as two kinds of aptamers have been employed for decorating these new mMOFs Fe_3_O_4_@UiO-66 for forming the signaling nano-probes. One of the aptamer protein nano-probe sandwich-type structures has been created in the presence of cTnI. Then, they employed nano-probes such as GQH DNAzyme, Fe_3_O_4_@UiO-66/Au@Pt NPs, as well as enzymes for catalysis of hydroquinone oxidation through hydrogen peroxide to amplify the electrochemical signals, at a working potential of −0.1 V (versus Ag/AgCl). The researchers observed the linear increase in the voltammetric signals in the concentration ranges from 0.01–100.0 ng·mL^−1^ cTnI and LOD equaled 5.7 pg·mL^−1^ [93].

**Figure 5:**
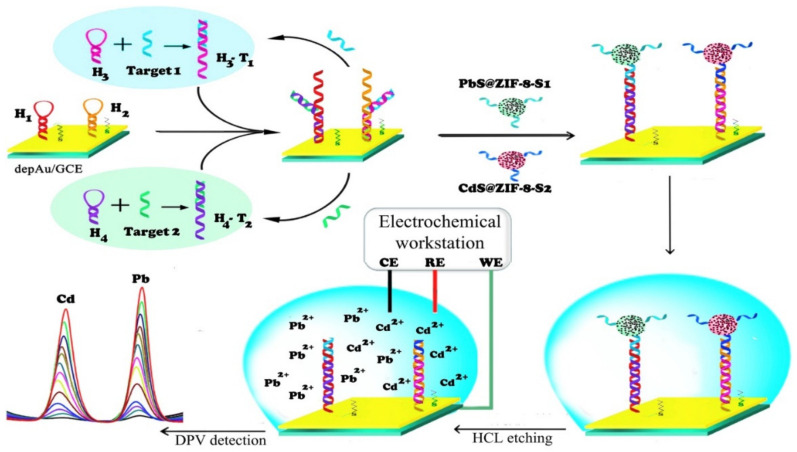
Construction of the electrochemical biosensor for simultaneous determination of miR-4521 and miR-1246. Reprinted with permission from Ref. [94].

Another study by Rezaei et al. designed an ultrasensitive electrochemical genosensor according to the Au NPs, PbS, and CdS QDs encapsulated ZIF-8 particles as the signal-amplifying tags, as well as a catalytic hairpin assembly (CHA) to detect simultaneously 2 hemophilia-related microRNAs (miR-4521 and miR-1246) (Figure 5). Rezaei and coworkers hybridized hairpins H1 and H2 by targeting miR-4521 (T2) and miR-1246 (T1) to form H2-T2 and H1-T1 duplex stranded DNAs (dsDNAs), which are capable of opening hairpins H3 and H4 to create H2-H4 and H1-H3 dsDNAs. Rezaei et al. also observed the release of the target for participation in another cycle to amplify the signals that formed multiple H1-H3 and H2-H4 dsDNAs. They employed the single-stranded fragments of H2-H4 and H1-H3 dsDNAs to hybridize CdS@ZIF-8-S2 and the PbS@ZIF-8-S1 for amplification of the electrochemical signals. In the next step, Cd (II) and Pb (II) ions have been released from the CdS@ZIF-8 and PbS@ZIF-8 tags via leaching HCI, which provided the ground for diagnosing the relative target miRs with DPV. After that, heavy metals quantum dots (QDs) have been encapsulated in ZIF-8 for producing the QDs@ZIF-8 multi-core-shell particles via the in situ growth of ZIF-8 in the presence of QDs. Due to the high growth of the quantities of the QDs tagged to each target miRs and because of a lot of QDs encapsulated in all QDs@ZIF-8 labels, the biosensor sensitivity with the particles of QDs@ZIF-8 as the signal tags have been nearly 15 times that of a biosensor that applied the QDs as the signal tags. Based on the optimal condition, the assay detected target miRs in ranges between 1 fM and 1 mM with the LOD equal to 0.28 fM and 0.19 fM for miR-4521 and miR-1246 (S/N = 3) [94].

Moreover, Li et al.s’ study reported an electrochemical approach according to the cascade primer exchange reaction (PER) with the MOF@Pt@MOF nanozyme for ultra-sensitive determination of exosomal miRNA. Target-stimulated PER with just a gated hairpin, DNA polymerase, and a primer may create a longer single strand autonomously and isothermally. In fact, protector B is released by the nascent strand that is employed for blocking the capture probes and binds the nanozyme to the sensing interface. Based on the nanozyme catalysis, H_2_O_2_ is decomposed into O_2_ and H_2_O and then creates an amplified electrochemical signal. This new biosensor takes advantage of cascade PER as well as higher catalytic activities of the multi-layered nanozyme and thus showed higher sensitivity with the LOD of 0.29 fM and higher specificity, which could differentiate between single-base mismatch and homologous miRNAs [95].

**Figure 6:**
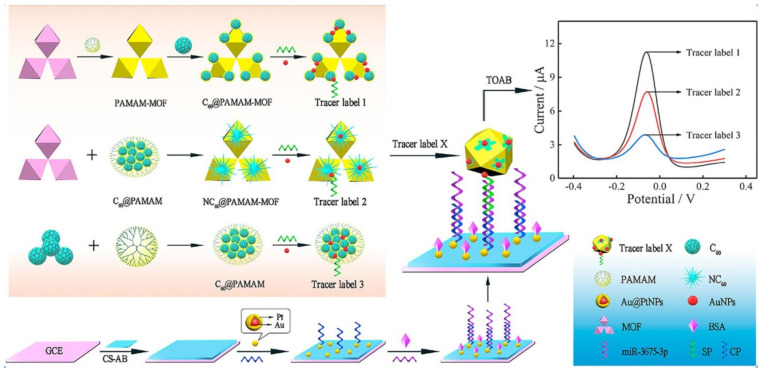
Preparation of 3 tracer labels and fabrication of the electrochemical miRNA biosensor for detecting miR-3675-3p. Reprinted with permission from Ref. [96].

Zuo et al. addressed the characterization of an electrochemical microRNA biosensor for detecting miR-3675-3p rapidly and sensitively according to numerous signal amplification methods. Fullerenes were doped with poly(amidoamine) (PAMAM)-functionalized MOF for forming a novel nano-hybrid of C_60_@PAMAM-MOF that exhibited greater redox activities than that of the other two synthesized C_60_-based nanohybrids in case of stimulation by tetraoctylammonium bromide (TOAB). Results also showed the larger specific surface area and considerable amino groups of C_60_@PAMAM-MOF for anchoring the Au NPs to immobilize the signal probe (SP) for forming the tracer label and enhancing the electrochemical response signals. Researchers have also observed the absorption of the core@shell AuPt NPs on CH-acetylene black (CS-AB) for acting as a sensing platform that could enhance the electron transfer and loading of the capture probe. Figure 6 demonstrates the construction of the miRNA biosensor and compares various tracer labels. Based on the optimal condition, this new biosensor displayed wider linear ranges for miR-3675-3p between 10.0 fM and 10.0 nM, with a LOD of 2.99 fM [96].

Gupta et al. focused on a copper-MOF composite, namely Cu_3_(BTC)_2_ with polyaniline (PANI) for preparing an impedimetric sensor for cTnI; therefore, they coated the composite of solvothermally synthesized Cu_3_(BTC)_2_/PANI as a thin layer on the screen-printed electrode (SPE) and conjugated the obtained electroconductive thin film with anti-cTnI antibodies. According to them, this sensor resulted in the impedimetric determination of cTnI antigen in the clinically significant concentration ranges from 1 to 400 ng mL^−1^ and they ended the antigen analysis within 5 min [97].

Biswas et al. reported the synthesis of Zr-trimesic acid MOF (MOF-808) nanocomposite with carbon nanotubes (CNTs) via in situ formation of MOF-808 over the activated CNT. MOF-808 is of a compatible nature and has a higher surface area and electro-catalytic abilities; therefore, researchers employed it for constructing an immuno-sensor for ultralow determination levels of the ovarian cancer biomarkers, carbohydrate antigen 125 (CA 125). They also used a GCE modified with MOF-808/CNT as one of the platforms for fabricating a label-free electrochemical immunosensor. Then, streptavidin has been functionalized for enriching the binding sites of the antibody of MOF-808/CNT. Finally, Biswas et al. observed LOD of 0.5 pg·mL^−1^ (S/N 3) and two linear detection ranges of 0.001−0.1 and 0.1−30.0 ng·mL^−1^ in this new immunosensor [98].

Li et al. constructed CuBTC@ molybdenum disulfide (CuBTC@MoS_2_) nanocomposite by a hydrothermal method to increase the electron- and ion-transfer capability. Then, AuNPs were electro-reduced on a CuBTC@MoS_2_-modified electrode by linear sweep voltammetry (LSV) for amplification of the connection between CA125 antibodies (CA125 Ab) and the CuBTC@MoS_2_-modified electrode. Because of the synergistic effect of CuBTC@MoS_2_ and AuNPs, the biosensor presented superior electrochemical efficiency. Subsequently, CuBTC@MoS_2_-AuNPs/CA125 Ab-modified electrodes were applied for the determination of the ovarian cancer biomarker CA125 in linear detection range between 0.5 mU mL^−1^ and 500.0 U mL^−1^ by DPV [99].

### 4.3. Detection of Small Biomolecules

It is well-known that small biomolecules are organic compounds with a low molecular weight that help the regulation of the biological processes of the body as well as the development of current treatments such as small molecule antigenomic treatments. Researchers have found that abnormal content of such compounds in the human body can lead to diverse diseases. As an example, glucose is involved in the process of metabolism and has been introduced as one of the main biomolecules to supply energy for bodies. Nonetheless, the excess or inadequate content of glucose would result in hypoglycemia or hyperglycemia [100,101]. For this reason, it is of high significance to determine these biomolecules sensitively and rapidly to diagnose and treat the above conditions. Table 3 summarizes several related investigations reporting the construction of the MOF-based electrochemical biosensors to detect these compounds.

Research conducted by Ruiqi et al. demonstrated the construction of one of the enzyme-free biosensors according to the porous MOF nano-composites; that is, core–shell structured Au@NC@GC nano-composites to detect dopamine (DA) and uric acid (UA) simultaneously. Then, they prepared Au@ZIF-8@ZIF-67 using a seed-mediated growth approach and carbonized it under a nitrogen atmosphere for synthesizing a nano-porous hybrid carbon material (Au@NC@GC). According to CV and DPV, the as-prepared Au@NC@GC modified (Au@NC@GC) GCE exhibited higher sensitivity and selectivity to determine DA and UA simultaneously. Researchers also observed a wider linear response for DA and UA in ranges between 10.0–150.0 μM and 10.0–600.0 μM, with the LOD equal to 0.746 nM and 0.773 nM (S/N = 3) [102].

In a related study, Choi et al. designed an electrochemical biosensor consisting of cellobiose dehydrogenase (CDH) and Co-hemin-MOF/chitosan (CH) composite to detect lactose. According to the analyses, Co-hemin MOF/CH composite displayed a considerable electrochemical function to detect lactose with fast responses (5 s) and higher sensitivity (102.3 µA mM^−1^ cm ^−2^). Lactose has also been detected by obtaining a lower LOD of 4.0 mM and a broader linear range between 10.0 and 100.0 mM. It is notable that the broader linear ranges reflected feasible dairy use of the biosensor based on the Co-hemin MOF electrode in their research [103].

Wang et al. dealt with the fabrication of a glucose sensor using a field-effect transistor (FET) with bimetallic nickel–copper (Ni/Cu) MOFs as its channel layers that have been enlarged in situ using a simplified one-step hydro-thermal approach and modified with GOx with the use of glutaraldehyde (GA) as the linker. As a result of the synergistic effects of the Cu and Ni ions in MOFs, the sensor (GOD-GA-Ni/Cu-MOFs-FET) exhibited an acceptable field-effect function and greater response to glucose via enzymatic reactions. The sensor also showed a piecewise linear association in the wider ranges between 1.0 μM and 20.0 mM), and reflected higher sensitivity (26.05 μAcm^−2^ mM^−1^) in lower concentrations of 1.0 to 100.0 μM and lower LODs of 0.51 μM [104].

In addition, Wang et al.s’ study addressed the implantation of the carboxyl MWCNTs into an Mn-based MOF (Mn-BDC) with the use of a one-step solvothermal approach; therefore, they constructed an electrochemical biosensor with the Mn-BDC@MWCNTs that exhibited broader concentration ranges and lower LODs to determine DA, UA, and ascorbic acid (AA) simultaneously with acceptable sensitivity and good selectivity. This biosensor successfully detected the materials with the wider linear detection range from 0.01 to 500.0, 0.02 to 1100.0, and 0.1 to 1150.0 μM for DA, UA, and AA and lower LODs of 0.002, 0.005, and 0.01 μM for DA, UA, and AA [105].

Li et al. developed an intelligent electrochemical biosensing system with a thermal self-regulation function based on an integration of phase-change microcapsules with a MOF based on zeolitic imidazolate framework-8. They first constructed a type of electroactive microcapsules, including a MOF-anchored polypyrrole/SiO_2_ double-layered shell and a phase-change material (PCM) core. These electro-active microcapsules were then applied to modify the surface of the electrode together with laccase as a bio-catalyst to fabricate a thermal self-regulatory biosensor. This biosensor shows better detection effectiveness for dopamine at higher temperatures. The biosensor also showed a linear range between 40.0 μM and 90.0 mM, and reflected higher sensitivity (3.541 μA L μmol^−1^ cm^−2^) with lower LOD of 0.0069 μM at 50 °C [106].

Zhang et al. described an amperometric biosensor for electrochemical detecting H_2_O_2_ and glucose under relatively low over-potential based on polyethyleneimine-functionalized MOF (P-MOF) supported AuNPs/nitrogen-doped graphene quantum dots (AuNPs/N-GQDs) and GOx. AuNPs/N-GQDs with high peroxidase mimicking activity was anchored on the P-MOF modified electrode surface as H_2_O_2_ sensors, exhibiting an excellent sensitivity (134.26 μA mM^−1^ cm^−2^) and a low LOD (3.38 μM). Subsequently, AuNPs/N-GQDs-P-MOF was applied as a nano-carrier for GOx to achieve “cascade nanoreactor” amplification for the determination of glucose. The amplified amperometric glucose biosensor presented reproducibility, superior anti-interference ability, and LOD 0.7 μM with excellent sensitivity (1512.4 μA mM^−1^cm^−2^). Moreover, an amperometric biosensor was applied for glucose detecting in human serum specimens with a recovery ranging from 93.2 to 99.3% [107].

### 4.4. Detection of Proteins

Multiple studies have proved the presence of proteins at diverse concentrations in the specimens of various sources. The detection of the protein concentration is of crucial importance. In case of the divergence of the activity of the proteins from their normal values, we would face several diseases such as cancer. Hence, analysis and measurement of the proteins in the biological specimens contributes importantly to the medical treatments, nutritional safety, health sector, and bioengineering [108,109,110]. It is possible to use various approaches to detect proteins. Researchers use the current electrochemical approaches for direct examinations of the proteins. Table 4 lists several studies reporting the construction of the MOF-based electrochemical biosensors to detect the proteins.

Zhou et al. provided one of the sensitive electrochemical impedimetric aptasensors using the cascade catalysis amplification driven by GOx for proteins (carcino-embryonic antigen, CEA as the tested model) with the use of the Cu-based MOFs functionalized with Pt NPs, hemin, GOx (Pt@CuMOFs-hGq-GOx), and aptamer. Then, CEA aptamer loaded on the Pt@Cu MOFs has been bound with hemin for forming hemin@G-quadruplex (hGq) by mimicking the activities of peroxidase. Via the sandwich-type reaction of the target CEA and CEA aptamers (Apt1 & Apt2), the produced Pt@CuMOFs-hGq-GOx as the signal transduction probes (STPs) has been captured to the interface of the modified electrode. Upon the introduction of glucose and 3,3 diaminobenzidine (DAB), the cascade reaction was started by GOx for catalyzing the glucose oxidation that in situ generated H_2_O_2_. At the same time, decomposing the resulting H_2_O_2_ has been highly enhanced by hGq and Pt@Cu MOFs as the synergistic peroxide catalysts, which has been followed by the oxidation process of DAB and creation of the non-conductive insoluble precipitates (IPs). Additionally, the electron transfer in the obtained sensing interface has been inhibited, and thus efficient amplification of the electrochemical impedimetric signal (EIS) has been observed; therefore, it could be said that higher sensitivity of this new CEA aptasensor enhanced with 0.023 pg mL^−1^ that is one of the excellent options to assay-specific clinical diseases associated with CEA [111].

**Figure 7:**
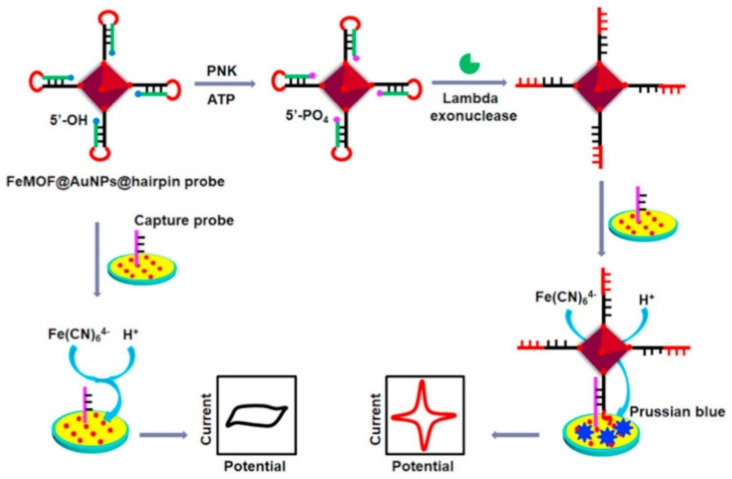
An electrochemical biosensor for PNK assay considering the greater quasi-reversible redox signal of Prussian blue established by the self-sacrificial label of FeMOF. Reprinted with permission from Ref. [112].

Cui et al. developed an electrochemical bio-sensor to detect the signals of T4 polynucleotide kinase (PNK) with regard to higher quasi-reversible redox signal of Prussian blue created by the self-sacrificial label of Fe-MOF. Researchers demonstrated the function of the DNA hairpin probe modified with the FeMOF@Au NPs at the 3′-thiol end as the PNK substrate. They found that the presence of PNK enabled 5′-phosphorylation of the hairpin probe that acted as the substrate of lambda exonuclease. In fact, Lambda exonuclease removed 5′ mononucleotide from the stem, unfolded the structure of the hairpin, and released the single-stranded DNA (ssDNA); therefore, the produced FeMOF@AuNP-modified ssDNA can specially hybridize with the capture probe for forming a double-strand DNA (dsDNA) duplex, which immobilizes Fe-MOF over the surface of the electrode. According to the analyses, the reaction of Fe^3+^ in the MOF with K_4_Fe (CN)_6_ formed the Prussian blue onto the surface of the electrode as well as the creation of a high electrochemical signal. Figure 7 displays the principle of the electrochemical biosensor to detect the PNK signals. Ultimately, their new electrochemical biosensor exhibited higher sensitivity with a LOD of 2.344 × 10^−4^ with the anodic peak current and 2.884 × 10^−4^ U mL^−1^ cathodic peak current as well as larger dynamic ranges between 0.0005 and 10 U mL^−1^ [112].

In their research, Wu et al. presented an electro-active Ni-MOF that has been assembled by the redox-active ligands 4,4′,4″-Tricarboxytriphenylamine (H_3_TCA) that is a triphenylamine derivative, as the electro-active source and magnetic ordered Ni_4_O_4_ clusters as the electronic transport nodes and employed for electrochemical aptasensing of thrombin (Tb). This Ni-MOF probe realized a sensitive, stable electrochemical signal output based on an easy sandwich-type aptasensing due to the periodic arrangement of the high-volume TCA active sites and acceptable magnetic ordered intermediate of Ni_4_O_4_ clusters in the regular porous structures of the MOFs. Finally, this new electrochemical aptasensor showed a broad linear association for Tb from 0.05 pM to 50.0 nM and a LOD of 0.016 pM (S/N = 3) [113].

Zhao et al. reliable and noninvasive electrochemical immunosensor for nuclear matrix protein 22 (NMP22) using the rGO-tetraethylenepentamine (TEPA) and gold NPs-platinum NPs-MOFs’ nanomaterials. They reported that rGO-TEPA elevated the loaded ability of the electrode and AuNPs-PtNPs-MOFs nanomaterials presented larger active sites to localize the target molecules, which largely enhanced the immunosensor sensitivity because of the greater biocompatibility, specific surface areas, chemical stability as well as conductivity. Considering suitable conditions, the new immuno-sensor exhibited a sensitive response toward NMP22 at the broader concentration ranges between 0.005 ng·mL^−1^ and 20.0 ng·mL^−1^, with a lower LOD equal to 1.7 pg·mL^−1^ (S/N = 3) [114].

**Figure 8:**
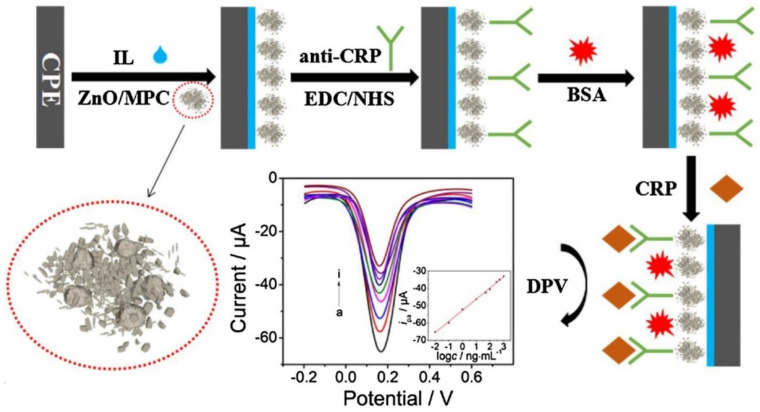
Preparation process of CRP immunosensor. Reprinted with permission from Ref. [115].

Dong et al. designed a new ZnO/porous carbon matrix (ZnO/MPC)-based electrochemical immunosensing by thermolyzing a mixed-ligand MOF (Zn-BDC-TED) to analyze the real samples of C-reactive protein (CRP). According to them, ionic liquid (IL) composite membrane modified electrode and ZnO/MPC showed considerable biocompatibility and conductivity for ultra-sensitive determination of CRP (see Figure 8). Then, CV and EIS have been used to monitor the step-by-step assembly process of the CRP immunosensor. When the parameter has been optimized, DPV response experienced a linear decline with the logarithm of CRP level in ranges from 0.01 to 1000.0 ng·mL^−1^ and LOD of 5.0 pg·mL^−1^ [115].

Using the Cu-MOF-rGO nanocomposite, Hatami et al. designed an electrochemical aptasensor for sensitively detecting Mucin 1 (MUC1), which is one of the large glycoproteins. They demonstrated the suitability of Cu-MOF-RGO as an option to immobilize MUC1 aptamer, as well as an electrochemical probe, exhibiting the regular peaks with acceptable reproducibility and stability. Then, researchers prepared the Cu-MOF-graphene oxide (Cu-MOF-GO) nano-composite and cast it on the surface of the electrode. For increasing the electrode conductivity, GO has been electrochemically reduced to rGO. They also observed a decline in the peak current of Cu in the nanocomposite in the presence of MUC1. This condition may be justified by forming the MUC1–aptamer complexes on the electrode, resulting in the blockage of the electron transfer of Cu at the electrode’s surface. Based on the optimal experimental condition, DPV showed a linear calibration curve in the concentration ranges between 0.1 pM and 10.0 nM (25.0 pg mL^−1^ and 2500.0 ng mL^−1^) with a LOD of 0.033 pM (7.5 pg mL^−1^) of MUC1 [116].

**Figure 9:**
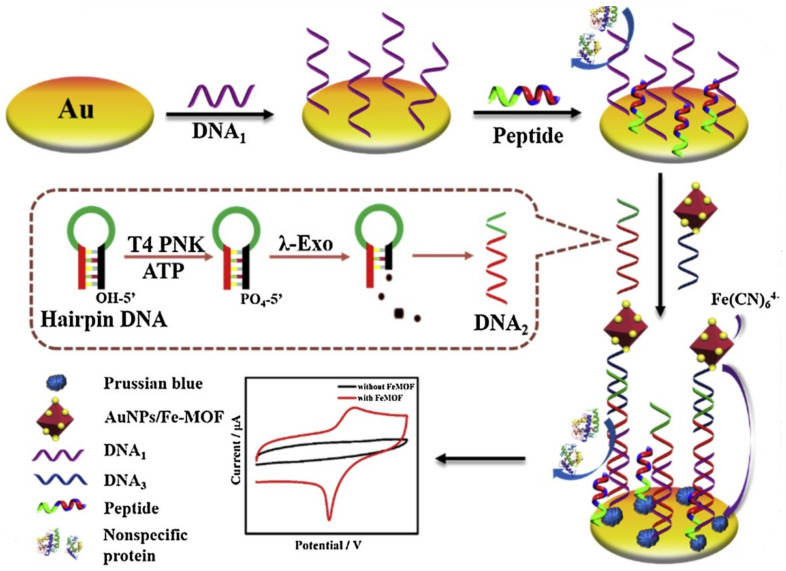
Construction procedure of the T4 PNK sensing platform. Reprinted with permission from Ref. [117].

Song et al. devised a sensitive, anti-fouling electrochemical enzyme sensing platform regarding a self-designed zwitterionic peptide and sacrificial iron (Fe) MOF (see Figure 9). This zwitterionic peptide is hydrophilic and has a neutral charge. Researchers applied the peptide for improving the antifouling capacity of the sensing tool; therefore, Fe-MOF with the amine groups has been loaded with the Au NPs and functionalized with a particular DNA sequence, and employed as a sacrificial label. Analyses have shown the ability of the sensing target, T4 polynucleotide kinase (PNK), for catalyzing 5′-hydroxyl termini of a certain hairpin DNA to the phosphate group. It has been found that hairpin DNA could be hydrolyzed by lambda exonuclease (λ-Exo) and released a single nucleotide. Results also observed the stimulation of a sandwich-type hybridization reaction in the presence of the released DNA sequence and selective attachment of the DNA bound Fe-MOF to the electrode surface, which may have a reaction with K_4_Fe (CN)_6_ for creating the electro-active Prussian blue and generation of the larger current responses. According to the anti-fouling peptide and sacrificial Fe-MOF, this new biosensor displayed acceptable analytical functions to determine T4 PNK, which had wider linear ranges between 1.0 × 10^−3^ and 10.0 U·mL^−1^ and a lower LOD of 3.5 × 10^−4^ U·mL^−1^ [117].

Tian et al. fabricated an electrochemical dual-aptamer biosensor based on the MOFs MIL-53(Al) decorated with Au@Pt NPs and enzymes for determination of SARS-CoV-2 nucleocapsid protein (2019-nCoV-NP) by co-catalysis of the nanostructures, G-quadruplex DNAzyme, and HRP. First, the two thiol-modified aptamers (N48 and N61) were immobilized on the surface electrode to capture the biomarker 2019-nCoV-NP. Then, the nanocomposites Au@Pt/MIL-53 (Al) were decorated by HRP and hemin/G-quadruplex DNAzyme as signal nanoprobe. The nanoprobe was used to amplify the biosensor signal by co-catalyzing the oxidation of hydroquinone in the presence of H_2_O_2_. Finally, the aptamer-protein-nanoprobe sandwich electrochemical sensing platform was constructed on the work electrode surface. This aptasensor has been employed for quantification of 2019-nCoV-NP with the wider linearity ranges between 0.025 ng/mL and 0.0 ng/mL and a lower LOD of 8.33 pg/mL [118].

### 4.5. Detection of Pathogens

It is widely accepted that pathogens are infectious agents, which lead to some diseases. These pathogens have been demonstrated to be microorganisms such as bacteria and fungi and molecular-scale infectious agents such as viruses. In fact, they have a widespread distribution in the foodstuff, marine and estuarine water, environment, the intestinal tract of animal, human, and soil. According to estimations, infectious diseases are due to ~40% of nearly 50 million total annual deaths. The waterborne pathogens lead to 10 to 20 million deaths, and more than 200 million people undergo nonfatal infections annually. Research approaches for fast, sensitive determination of the pathogens in the complicated matrices such as aerosols and body fluids, as well as over the surface, have been crucial for treating infectious diseases and preventing the infection prevalence [119,120,121]. Table 5 reports several related studies on the construction of the MOF-based electrochemical biosensors to detect pathogens.

Considering the previous research [122,123], MPT64 has been shown to be one of the kinds of 24-kDa protein that is just secreted by Mycobacterium tuberculosis (MTB) in the initial and middle period of bacterial growth. Hence, it is possible to utilize the protein for specific early determination of MTB.

Li et al. characterized one of the ultra-sensitive aptasensors for voltammetric detection of the Mycobacterium tuberculosis antigen MPT64 in the human sera. Researchers, synthesized an amino-modified Zr(IV) based MOF (UiO-66-NH_2_, consisting of Zr_6_O_32_ units and 2-amino-terephthalate linkers) with the higher specific surfaces and employed them as the carrier of the gold NPs and aptamers. In the next step, they constructed the signaling nano-probe following the casting of HRP on the nano-materials. Then, two aptamers with the synergistic effect on the binding of MPT64 have been anchored on the gold electrode. According to DPV, researchers observed the greatest peak current when the ratio of both aptamers was 1:1. Finally, the assay showed a wider range of linear response (between 0.02 and 1000 pg·mL^−1^ of MPT64) and a LOD of 10.0 fg·mL^−1^ at a working potential of nearly −96 mV (versus Ag/AgCl) [124].

Additionally, ESAT-6, which has a low-molecular-weight secreted protein, has been separated from the filtrate of the short-term MTB culture. ESAT-6 emerges initially in case of the infection of an organism with MTB, and thus it importantly contributes to the initial diagnosis of tuberculosis. Hence, it is possible to use ESAT-6 as an acceptable alternative to detect MTB.

According to Li et al., a voltammetric aptasensor has been characterized for ultra-sensitive determination of ESAT-6 antigen; therefore, researchers deposited rGO doped with MOF onto a GCE, which increased immobilization of the electro-active Toluidine Blue (TB) and simplified the electron transfer from TB to the modified GCE. They applied platinum/gold core/shell (Pt@Au) NPs for assembling the thiolated ESAT-6 binding aptamer (EBA) over a modified electrode and amplifying responses to TB. This modified GCE that has been acted at −0.36 V (vs. SCE) linearly responded in 1.0 × 10^−4^ to 2.0 × 10^2^ ng⋅mL^−1^ ESAT-6 concentration range, and LOD for ESAT-6 equaled 3.3 × 10^−5^ ng⋅mL^−1^ [125].

**Figure 10:**
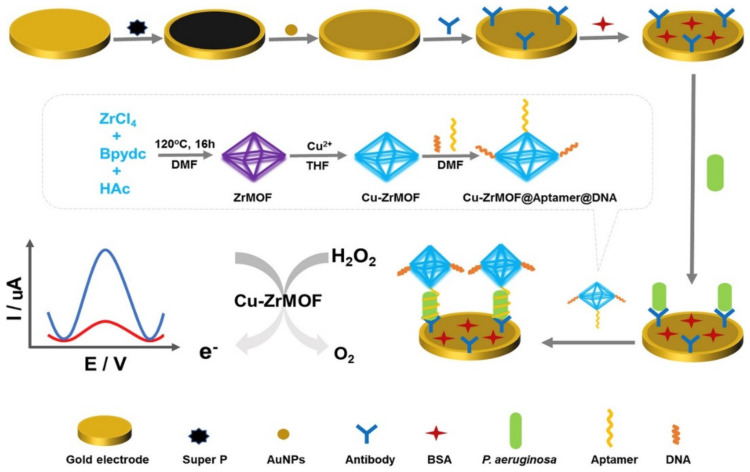
Electrochemical detection of *P. aeruginosa*. Reprinted with permission from Ref. [126].

Zhang et al. made their enzyme-free electrochemical biosensor for sensitive, fast detection of *Pseudomonas aeruginosa* (*P. aeruginosa*); therefore, they synthesized ZrMOF with a large surface area that offered very good absorption. Then, it has been linked to a certain content of Cu^2+^ for Cu-ZrMOF synthesis with higher catalytic activities. In the next step, Cu-ZrMOF@Aptamer@DNA nano-composite has been formed and applied as a signal probe for catalyzing H_2_O_2_ decomposition. After that, higher conductive Super-P was entered for increasing the electron transfer to obtain acceptable detection sensitivity. Figure 10 demonstrates the construction procedure and assay principle. This new biosensor has been employed for quantification of *P. aeruginosa* with wider linearity ranges between 10.0 and 10^6^ CFU mL^−1^ and lower LOD of 2.0 CFU mL^−1^ (S/N = 3) [126].

Moreover, Gupta addressed the Cu-MOF-based electrochemical biosensor for largely sensitive determination of *E. coli* bacterium. For realizing an MOF-based electrochemically active platform, researchers mixed Cu_3_(BTC)_2_ (BTC=1,3,5-benzenetricarboxylic acid) with polyaniline (PANI). Then, thin films of Cu_3_(BTC)_2_-PANI, over the indium-tin-oxide (ITO) substrate, have been biointerfaced with anti-*E. coli* antibodies to be used as one of the new biosensing electrodes. According to EIS of the signal measurements, this sensor displayed higher sensitivity for detecting the very low concentration of *E. coli* (2 cfu/mL) in a short response period (~2 min) that has been selective in the presence of other nonspecific bacteria [127].

Wang et al. provided their specific electrochemical aptasensors for fast on-site determination of vibrio parahaemolyticus (V.P) in seafood; therefore, they used magnetic nanoscale MOFs (Fe_3_O_4_@NMOF) with the labeled aptamer against V.P as capture probes, whereas gold NPs in combination with ferrocene and phenylboronic acid functioned as the nano-labels. In the case of V.P detection, researchers observed the formation of the sandwich-type complex of capture probe V.P enanolabel and magnetic attachment to an SPE to measure the signals. Based on the optimized condition, the V.P amount can be assessed by increasing the ferrocene electrochemical signals. The concentration ranged between 10 and 10^9^ cfu/mL. Wang et al. found that it is possible to achieve fast on-site assays with the use of the compact SPE with a LOD of 20 min as the measurement platform [128].

Furthermore, Panhwar et al. presented a dual-responsive disposable electrode to enumerate *Escherichia coli* K12 (*E. coli* K12) using an immunomagnetic separation method that showed the bacterial detection in the whole blood. They applied the citrate-capped gold NPs and modified MOFs as spectro-electrochemical and capture probe labels. Moreover, they utilized CV for *E. coli* quantification in ranges between 10^1^ and 10^7^ cfu/mL with a LOD of 1.0 cfu/mL [129].

Detecting the staphylococcus aureus specific gene when combined with *mec*A gene has been shown to be highly crucial for accurately identifying the methicillin-resistant *Staphylococcus aureus* (MRSA). The study conducted by Dai et al. dealt with the fabrication of a homogeneous electrochemical DNA sensor to simultaneously detect *nuc* and *mec*A gene in MRSA. Researchers utilized MOF (type UiO-66-NH_2_) as the nano-carrier and encapsulated electro-active dyes, epirubicin (EP), and methylene blue (MB) in UiO-66-NH_2_, which have been locked by the hybrid double-stranded DNA. Regarding the target response electro-active dye release, in case of the presence of the target DNA, it thoroughly hybridized by displacing DNA (DMB and DEP). Then, DMB and DEP are displaced from the surfaces of MOFs, which result in releasing electro-active dyes. Co-Zn bimetallic ZIF-derived N-doped porous carbon is used to amplify electrodes for improving electro-catalytic sensitivity and function. Finally, DPV peak current of EP and MB has been exhibited at −0.14 V to −0.53 V vs. the Ag/AgCl reference electrode. Based on the optimum condition, LOD of *nuc* and *mec*A genes equaled 1.6 fM and 3.7 fM [130].

Consequently, Liu et al. illustrated the description of one of a sandwich-type electrochemical immunosensors for avian leukosis virus subgroup J (ALV-J) and thus provided immuno-sensor via modification of a GCE with rGO functionalized with magnetite (Fe_3_O_4_) NPs and tannic acid. In the next step, they deposited the primary antibodies (Ab1) onto a modified GCE and applied hollow ZIF (eZIF) crystals functionalized with tannic acid (TA) and carrying secondary antibodies (Ab2) and HRP to amplify the signals. They referred to the fact that hollow eZIF crystals can be one of the very good carriers for HRP and Ab2. Based on the optimum condition, immunoassay exhibited a LOD ranging between 152 and 10,000 TCID50 mL^−1^ (TCID50 represents the 50% tissue culture infective dose) and a lower LOD of 140 TCID50 mL^−1^ [131].

**Figure 11:**
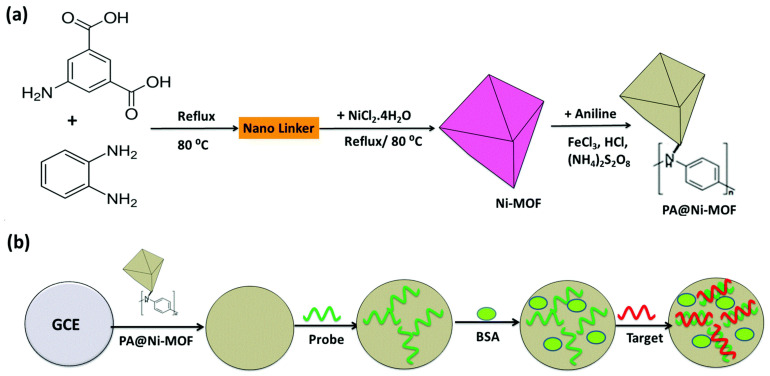
(**a**) PA@Ni-MOF composite synthesis via chemical oxidation polymerization of the aniline monomer in the presence of Ni-MOF. (**b**) Construction of the electrochemical HCV-nucleic acid biosensor. Reprinted with permission from Ref. [132].

Sheta et al. presented their new label-free electrochemical biosensor with a polyaniline@nickel metal–organic framework (Ni-MOF) nanocomposite to directly detect the unamplified Hepatitis-C virus ribonucleic acid (HCV-RNA); therefore, they constructed a biosensor with a uniform layer-by-layer deposition of polyaniline@Ni-MOF nano-composite, BSA, and deoxyribonucleic acid (DNA) probe over a GCE and monitored it with real-time CV and EIS. Figure 11a,b depict the synthesis process of the PA@Ni-MOF nanocomposite and fabrication of the electrochemical HCV biosensor. According to their research, this new biosensor has been largely effective for quantitative sensing of the HCV target in the presence of non-specific nucleic acids in ranges between 1 fM and 100 nM with a LOD of 0.75 fM at an S/N ratio of 3 [132].

Jia et al. developed electrochemical biosensors based on AgNP embedded polymer–zirconium-based MOF (polyUiO-66@AgNPs) to the determination of respiratory viruses, inclusive influenza A (H1N1) virus and the N-gene of severe acute respiratory syndrome coronavirus 2 (SARS-CoV2). The polyUiO-66@AgNPs was synthesized by a 1,4-benzenedicarboxylic acid ligand as the linker and AgNPs as the dopants by using a hydrothermal reaction. A large number of H1N1-targeted antibodies (namely Ab/polyUiO-66@AgNPs) and N-gene-targeted aptamers (namely Apt/polyUiO-66@AgNPs) can be singly adsorbed on the substrate modified by integrating the enhanced electrochemical activity and biocompatibility of AgNPs with the great pore size, wide surface area, and rich functionality of polyUiO-66. As a result, the polyUiO-66@AgNP-based aptasensor displays very low LODs of 54.7 and 49.4 fg mL^−1^ to H1N1 in the dynamic range between 0.1 pg mL^−1^ and 1.0 mg mL^−1^ as deduced by EIS and DPV, respectively, whiles the proposed biosensor exhibits LODs of 23.4 and 18.2 fg/mL for determination of SARS-CoV-2 N-gene in the linear range between 0.1 pg/mL and 1.0 ng/mLvia EIS and DPV, respectively [133].

## 5. Conclusions and Future Directions

This review quantified the proteins, disease biomarkers, pathogens, and small biomolecules in biological specimens on the respective data on the seriousness of specific diseases. Reliability and sensitivity have been shown to be an important challenge because of their ultra-low amounts. Moreover, we showed the limit of detection of electrochemical biosensors such as analytes. Results also indicated the prominence of designing the mentioned sensors for the initial diagnosis of diseases for clinical therapies, treatment, the discovery of preventing approaches, and disease monitoring. It has been found that MOFs’ materials may be innovative biosensing options for electrochemical sensing uses; therefore, MOF significantly contributes to the establishment of innovative biosensors to detect biomedical analytes due to several specific physiochemical features such as higher porosity, larger volumes of the pores, larger surface areas, acceptable biocompatible and adjustable structures and chemical functionality.

Studies on the MOF-based biosensors are undergoing their early days because a limited number of MOF structures contribute to electrochemical biosensing. For the additional expansion of its uses and enhancement of the sensing function, several dimensions must be researchers. Firstly, studying the synthesis-property-use association of the MOF-based substances for electrochemical biosensing and designing novel synthesis methods for preparing substances with the intended features would certainly indicate theoretical prominence and functional values for fast expansion of such an interesting area. Secondly, most MOFs have been shown to be semiconductors or insulators. Hence, fabrication and conductivity of the redox-active of MOFs are a major issue for MOF-based electrochemical biosensors; therefore, MOFs combined with the conductive functional samples such as carbon nanomaterials or metal NPs for promoting the smooth distribution of more active sites would be the other functional strategy for obtaining higher sensitivity. Thirdly, for obtaining an electrochemical sensor with the intended sensitivity, one of the efficient methods would be to design nano-scale MOFs with specific structures such as two-dimensional MOF for improving the electro-active area of the electrode and promoting mass and or electron transfer efficiency. In addition, immobilization of two-dimensional MOF of the electro-active samples must be greater than that of the bulk MOFs due to higher accessible postmodification sites of 2D MOF. Fourthly, another useful technique for improving the electrochemical functions of the MOFs’ composite would be to utilize MOF as the template for the synthesis of metal oxide or carbon nanocomposites. Fifthly, if numerous new approaches are combined, essential technological advancements would be largely possible. As an instance, presenting novel nanomaterials such as covalent organic frameworks (COFs), supra-molecular hydrogels (SMGs), and metal–organic gels (MOGs) as proper matrices for realizing a very good distribution of the MOFs would be largely interesting. Furthermore, MOF@COFs and core-shell MOF@MOF may provide attractive features to enhance the sensing uses. Generally, using the MOF-based substances in electrochemical biosensing has not been so mature, and thus innovative studies can be remarkably conducted in this field. Finally, in case of an acceptable development and post-modification, MOFs-based materials may be more proper as one of the signal transduction platforms for electrochemical biosensing with diverse uses in clinical diagnosis and disease monitoring.

## Figures and Tables

**Table 1:** Different characteristics of the MOF-based electrochemical biosensors utilized to analyze pharmaceutical drugs.

Biosensor Materials	Analyte	Technique	Linear Range	Detection Limit	Ref.
anti-monensin antibodies and Zn/Ni-ZIF-8-800@grahene composites	Monensin	DPV	0.25–100.0 ng mL^−1^	0.11 ng mL^−1^	[77]
hemin@MOFs/AuPt-Ab2-HRP/HRP	Maduramicin	i-t	0.1–50.0 ng mL^−1^	0.045 ng mL^−1^	[78]
CD-CuMOF-CNF and artificial enzyme model	RR-ethambutol	SWV	1.0 × 10^−7^–1.0 × 10^−4^	3.10 × 10^−8^ M	[79]
	SS-ethambutol		5.0 × 10^−7^–2.5 × 10^−4^	8.52 × 10^−8^ M	
Cu-1	L-tyrosine	DPV	0.01–0.09 mM	5.822 μM	[80]
CoxNi_3-x_(HITP)_2_/Apt	Enrofloxacin	EIS	0.001–1.0 pg·mL^−1^	0.2 fg·mL^−1^	[81]
C_60_@UiO-66-NH_2_ and aptamer	Tobramycin	EIS	2.1 × 10^−3^–106.9 nM	0.377 pM	[82]

**Table 2:** Different characteristics of the MOF-based electrochemical biosensors for detecting the disease biomarkers.

Biosensor Materials	Biomarker	Technique	Linear Range	Detection Limit	Ref.
N-GNRs-Fe-MOFs@AuNPs-Ab_1_ and AuPt-MB-Ab2	Galectin-3	DPV	100.0 fg mL^−1^–50.0 ng mL^−1^	33.33 fg mL^−1^	[87]
PdNPs@Fe-MOFs/SA/SPs and hairpin assembly	miR-122	Amperometric	0.01 fM–10.0 pM	0.003 fM	[88]
rGO-SnO_2_/HNMs/AuPt and SiO_2_-Ab_2_	Lymphocyte activation gene-3	Amperometric	0.01 ng·mL^−1^–1.0 μg·mL^−1^	1.1 pg·mL^−1^	[89]
Ab2-functionalized GOx-Cu-MOF	carbohydrate antigen 15–3	Impedimetric	10.0 μU/mL–100.0 U/mL	5.06 μU/mL	[90]
Fe_3_O_4_@UiO-66/Cu@Au nanocomposites and NTH-Tro4 + Tro6	Cardiac troponin I	DPV	0.05–100.0 ng/mL	16.0 pg/mL	[91]
Invertase/CP/Au/CuMOF and hairpin probe 1 & 2	DNA methyltransferase	DPV	0.002-12.0 U mL^−1^	0.001 U mL^−1^	[92]
Fe_3_O_4_@UiO-66/Au@PtNP nanocomposites and GQH DNAzyme	Cardiac troponin I	DPV	0.01–100.0 ng·mL^−1^	5.7 pg·mL^−1^	[93]
Au nanoparticles, PbS and CdS QDs encapsulated ZIF-8 particles and catalytic hairpin assembly	miR-1246	DPV	1.0 fM–1.0 mM	0.19 fM	[94]
	miR-4521		1.0 fM–1.0 mM	0.28 fM	
MOF@Pt@MOF nanozyme	Exosomal miRNA	DPV	1.0 fM–1.0 nM	0.29 fM	[95]
C_60_@PAMAM-MOF coupled with CS-AB and Au@PtNPs	miR-3675-3p	DPV	10.0 fM–10.0 nM	2.99 fM	[96]
b/Cu_3_(BTC)_2_/PANI	Troponin I	Impedimetric	1.0–400.0 ng mL^−1^	0.8 ng mL^−1^	[97]
MOF-808/CNT/Ab	carbohydrate antigen 125	DPV	0.001–30.0 ng·mL^−1^	0.5 pg·mL^−1^	[98]
CuBTC@MoS_2_-AuNPs/CA125 Ab	ovarian cancer biomarker CA125	DPV	0.5 mU/Ml–500.0 U/mL	0.0005 U/mL	[99]

**Table 3:** Different characteristics of the MOF-based electrochemical biosensors for detecting small biomolecules.

Biosensor Materials	Analytes	Technique	Linear Range	Detection Limit	Ref.
Au@NC@GC	Uric acid	DPV	10.0–600.0 μM	10.0–150.0 μM	[102]
	Dopamine		0.773 nM	0.746 nM	
Co-hemin MOF/chitosan/PcCDH	Lactose	Amperometric	10.0–100.0 mM	4.0 mM	[103]
GOD-GA-Ni/Cu-MOFs-FET	Glucose	Amperometric	1.0 μM–20.0 mM	0.51 μM	[104]
Mn-BDC@MWCNTs	Ascorbic acid	DPV	0.1–1150.0 μM	0.01 μM	[105]
	Dopamine		0.01–500.0 μM	0.002 μM	
	Uric acid		0.02–1100.0 μM	0.005 μM	
ZIF-8/PPy@SiO_2_-MEPCM and laccase	Dopamine	DPV	40.0–90.0 μM	0.0069 μM	[106]
GOx-AuNPs/N-GQDs-P-MOF	Glucose	Amperometric	0.02–10.0 μM	0.7 μM	[107]

**Table 4:** Different properties of the MOF-based electrochemical biosensors for detecting proteins.

Biosensor Materials	Proteins	Technique	Linear Range	Detection Limit	Ref.
Pt@CuMOFs-hGq-GOx	Carcinoembryonic antigen	Impedimetric	0.05 pg mL^−1^–20.0 ng mL^−1^	0.023 pg mL^−1^	[111]
FeMOF@AuNPs-hairpin	T4 polynucleotide kinase	CV	0.0005–10.0 U mL^−1^	2.344 × 10^−4^	[112]
AP II/AuNPs/Ni-MOF	Thrombin	DPV	0.05 pM–50.0 nM	0.016 pM	[113]
rGO-TEPA/AuNPs-PtNPs-MOFs	Nuclear matrix protein 22	DPV	0.005–20.0 ng·mL^−1^	1.7 pg·mL^−1^	[114]
anti-CRP/ZnO/MPC/IL	C-reactive protein	DPV	0.01–1000.0 ng·mL^−1^	5.0 pg·mL^−1^	[115]
Cu-MOF-GO and MUC1–aptamer	Mucin 1	DPV	0.1 pM–10.0 nM	0.033 pM	[116]
Fe-MOF/AuNPs/ DNA	T4 polynucleotide kinase	DPV	1.0 × 10^−3^–10.0 U·mL^−1^	3.5 × 10^−4^ U·mL^−1^	[117]
GQH DNAzyme/Dual-aptamer/HRP/Au@Pt/MIL-53	COVID-19 nucleocapsid protein	DPV	0.025–50.0 ng mL^−1^	8.33 pg mL^−1^	[118]

**Table 5:** Different properties of the MOF-based electrochemical biosensors presented for detecting pathogens.

Biosensor Materials	Pathogens	Technique	Linear Range	Detection Limit	Ref.
aptamer/HRP/AuNP/UiO-66-NH_2_	Mycobacterium tuberculosis antigen MPT64	DPV	0.02–1000.0 pg·mL^−1^	10.0 fg·mL^−1^	[124]
ESAT-6/BSA/EBA/Pt@Au/TB/P-MOF-rGO	Mycobacterium tuberculosis antigen ESAT-6	CV	1.0 × 10^−4^–2.0 × 10^2^ ng⋅mL^−1^	3.3 × 10^−5^ ng⋅mL^−1^	[125]
Cu-ZrMOF@Aptamer@DNA	*Pseudomonas aeruginosa*	DPV	10–10^6^ CFU mL^−1^	2.0 CFU mL^−1^	[126]
Ab/Cu_3_(BTC)_2_-PANI	*Escherichia coli*	Impedimetric	2.0–2.0 × 10^8^ cfu/mL	2.0 cfu/mL	[127]
Fe_3_O_4_@NMOF-Apt	*Vibrio parahaemolyticus*	SWV	10–10^9^ cfu/mL	3.0 cfu/mL	[128]
MOFs-Ab-*E. coli*-AuNP	*Escherichia coli* K12	CV	10^1^–10^7^ cfu/mL	1.0 cfu/mL	[129]
UiO-66/BMZIF-derived NPCs	*Mec*A gene in methicillin-resistant *Staphylococcus aureus*	DPV	5.0 × 10^−15^–1.0 × 10^−10^ M	3.7 fM	[130]
	*Nuc* gene in methicillin-resistant *Staphylococcus aureus*		5.0 × 10^−15^–1.0 × 10^−10^ M	1.6 fM	
rGO-TA-Fe_3_O_4_/ BSA/Ab1/ALV-J/eZIF-Ab2-HRP	Avian leukosis virus	DPV	152.0–10,000 TCID50 mL^−1^	140.0 TCID50 mL^−1^	[131]
polyaniline@Ni-MOF/DNA/BSA	Hepatitis-C virus	Impedimetric	1.0 fM–100.0 nM	0.75 fM	[132]
Ab/polyUiO-66@AgNPs	H1N1 virus	EIS	100.0–1.0 × 10^9^ fg mL^−1^	54.7 fg mL^−1^	[133]
		DPV	100.0–1.0 × 10^9^ fg mL^−1^	49.4 fg mL^−1^	
Apt/polyUiO-66@AgNPs	SARS-CoV2 virus	EIS	100.0–1.0 × 10^6^ fg mL^−1^	23.4 fg mL^−1^	
		DPV	100.0–1.0 × 10^6^ fg mL^−1^		

## Data Availability

Data reported in this study are available from the reference list.
